# Efficacy of awake prone positioning in patients with covid-19 related hypoxemic respiratory failure: systematic review and meta-analysis of randomized trials

**DOI:** 10.1136/bmj-2022-071966

**Published:** 2022-12-07

**Authors:** Jason Weatherald, Ken Kuljit S Parhar, Zainab Al Duhailib, Derek K Chu, Anders Granholm, Kevin Solverson, Kimberley Lewis, Morten Hylander Møller, Mohammed Alshahrani, Emilie Belley-Cote, Nicole Loroff, Edward T Qian, Cheryl L Gatto, Todd W Rice, Dan Niven, Henry T Stelfox, Kirsten Fiest, Deborah Cook, Yaseen M Arabi, Waleed Alhazzani

**Affiliations:** 1 Department of Medicine, Division of Pulmonary Medicine, University of Alberta, Edmonton, AB, Canada; 2 Department of Medicine, Division of Respirology, University of Calgary, Calgary, AB, Canada; 3 Libin Cardiovascular Institute, University of Calgary, Calgary, AB, Canada; 4 Department of Critical Care Medicine, University of Calgary and Alberta Health Services, Calgary, AB T2N 5A1, Canada; 5 O’Brien Institute for Public Health, Calgary, AB, Canada; 6 Critical Care Medicine Department, King Faisal Specialist Hospital and Research Centre, Riyadh, Saudi Arabia; 7 Department of Medicine, McMaster University, Hamilton, ON, Canada; 8 Department of Health Research Methods, Evidence, and Impact, McMaster University, Hamilton, ON, Canada; 9 The Research Institute of St Joe’s Hamilton, Hamilton, ON, Canada; 10 Department of Intensive Care, Copenhagen University Hospital–Rigshospitalet, Copenhagen, Denmark; 11 Department of Medicine, Division of Critical Care, McMaster University, Hamilton, ON, Canada; 12 Department of Emergency and Critical Care, Imam Abdulrahman Bin Faisal University, Dammam, Saudi Arabia; 13 Population Health Research Institute, Hamilton, ON, Canada; 14 Knowledge Resource Service, Alberta Health Services, Edmonton, AB, Canada; 15 Division of Allergy, Pulmonary, and Critical Care Medicine, Vanderbilt University Medical Center, Nashville, TN, USA; 16 Vanderbilt Institute for Clinical and Translational Research, Vanderbilt University Medical Center, Nashville, TN, USA; 17 Department of Biostatistics, Vanderbilt University Medical Center, Nashville, TN, USA; 18 College of Medicine, King Saud Bin Abdulaziz University for Health Sciences, Riyadh, Saudi Arabia; 19 King Abdullah International Medical Research Center, Riyadh, Saudi Arabia; 20 King Abdulaziz Medical City, Ministry of National Guard Health Affairs, Riyadh, Saudi Arabia; 21 Department of Critical Care, College of Medicine, King Saud University, Riyadh, Saudi Arabia; * Contributed equally

## Abstract

**Objective:**

To determine the efficacy and safety of awake prone positioning versus usual care in non-intubated adults with hypoxemic respiratory failure due to covid-19.

**Design:**

Systematic review with frequentist and bayesian meta-analyses.

**Study eligibility:**

Randomized trials comparing awake prone positioning versus usual care in adults with covid-19 related hypoxemic respiratory failure. Information sources were Medline, Embase, and the Cochrane Central Register of Controlled Trials from inception to 4 March 2022.

**Data extraction and synthesis:**

Two reviewers independently extracted data and assessed risk of bias. Random effects meta-analyses were performed for the primary and secondary outcomes. Bayesian meta-analyses were performed for endotracheal intubation and mortality outcomes. GRADE certainty of evidence was assessed for outcomes.

**Main outcome measures:**

The primary outcome was endotracheal intubation. Secondary outcomes were mortality, ventilator-free days, intensive care unit (ICU) and hospital length of stay, escalation of oxygen modality, change in oxygenation and respiratory rate, and adverse events.

**Results:**

17 trials (2931 patients) met the eligibility criteria. 12 trials were at low risk of bias, three had some concerns, and two were at high risk. Awake prone positioning reduced the risk of endotracheal intubation compared with usual care (crude average 24.2% *v* 29.8%, relative risk 0.83, 95% confidence interval 0.73 to 0.94; high certainty). This translates to 55 fewer intubations per 1000 patients (95% confidence interval 87 to 19 fewer intubations). Awake prone positioning did not significantly affect secondary outcomes, including mortality (15.6% *v* 17.2%, relative risk 0.90, 0.76 to 1.07; high certainty), ventilator-free days (mean difference 0.97 days, 95% confidence interval −0.5 to 3.4; low certainty), ICU length of stay (−2.1 days, −4.5 to 0.4; low certainty), hospital length of stay (−0.09 days, −0.69 to 0.51; moderate certainty), and escalation of oxygen modality (21.4% *v* 23.0%, relative risk 1.04, 0.74 to 1.44; low certainty). Adverse events related to awake prone positioning were uncommon. Bayesian meta-analysis showed a high probability of benefit with awake prone positioning for endotracheal intubation (non-informative prior, mean relative risk 0.83, 95% credible interval 0.70 to 0.97; posterior probability for relative risk <0.95=96%) but lower probability for mortality (0.90, 0.73 to 1.13; <0.95=68%).

**Conclusions:**

Awake prone positioning compared with usual care reduces the risk of endotracheal intubation in adults with hypoxemic respiratory failure due to covid-19 but probably has little to no effect on mortality or other outcomes.

**Systematic review registration:**

PROSPERO CRD42022314856.

## Introduction

Patients with covid-19 can develop hypoxemic respiratory failure, potentially necessitating admission to hospital for supplemental oxygen or to an intensive care unit (ICU) for mechanical ventilation.^1^ ^2^ ^3^ Although most patients have mild disease, some will develop severe disease, including acute respiratory distress syndrome.^2^ Interventions aimed at limiting illness severity and reducing the need for invasive mechanical ventilation are needed.

Non-pharmacological interventions such as prone positioning are life saving for patients with moderate-severe acute respiratory distress syndrome receiving mechanical ventilation.^4^ ^5^ ^6^ Although high certainty evidence exists for the use of prone positioning in patients receiving invasive ventilation for non-covid-19 related acute respiratory distress syndrome,^5^ ^6^ it is unclear whether awake prone positioning improves outcomes in spontaneously breathing non-intubated patients with covid-19. Previous systematic reviews and meta-analyses of observational studies suggested that awake prone positioning was associated with improved oxygenation and low endotracheal intubation rates.^7^ ^8^ ^9^ ^10^ Despite these outcomes, the tolerability, safety, and efficacy of awake prone positioning remains unclear in patients with covid-19 related hypoxemic respiratory failure. A prospective meta-analysis of six individual randomized controlled trials reported a reduction in the risk of treatment failure (ie, a composite outcome of intubation or death) and a reduction in the risk of endotracheal intubation. The results of this prospective meta-analysis must be interpreted cautiously as the effect was probably driven by one of the included randomized controlled trials.^11^ Two recent systematic reviews and meta-analyses had limitations, such as being driven by the results of the prospective meta-analysis^12^ or combining both observational and randomized studies.^10^ Moreover, a comprehensive systematic review on awake prone positioning in patients with covid-19 that also incorporates recent trials is needed.

Given the uncertainty about the clinical benefits of awake prone positioning^13^ and recent evidence from three trials with more than 900 additional patients,^14^ ^15^ ^16^ we performed a systematic review and meta-analysis. We used both frequentist and bayesian methods to evaluate the efficacy and safety of awake prone positioning compared with usual care in trials of non-intubated adults with hypoxemic respiratory failure due to covid-19.

## Methods

We conducted this systematic review and meta-analysis according to the Cochrane Handbook for Systematic Reviews of Interventions,^17^ adhered to the Preferred Reporting Items for Systematic Review and Meta-analysis (PRISMA) (see supplemental eMethods 1),^18^ and prospectively registered the protocol on PROSPERO.

### Search strategy and study selection

The Cochrane Central Register of Controlled Trials, Embase, and Medline were systematically searched from inception to 4 March 2022. We also searched the preprint server medrxiv for relevant unpublished studies and ClinicalTrials.gov for ongoing or recently completed trials. The reference lists of included studies were reviewed for any additional eligible studies. A medical librarian designed the search strategy for all databases. A second medical librarian subsequently and independently reviewed the search strategy.^19^ The search terms are available in supplemental eMethods 2.

Two reviewers independently, and in duplicate, screened the list of titles and abstracts. Reviewers assessed the full texts of potentially eligible studies. To be eligible for inclusion the studies needed to use a randomized controlled trial design, including cluster randomized controlled trials and quasi-randomized controlled trials using the Cochrane suggested definitions of these study types^17^ ^20^ ^21^; include hospital patients with hypoxemic respiratory failure due to covid-19; compare awake prone positioning with usual care (no prone positioning); and report on at least one of the outcomes of interest. Reviewers excluded non-randomized studies.

### Outcomes

The primary outcome was endotracheal intubation at the longest time point reported. Secondary outcomes included mortality at the longest reported interval, hospital length of stay, ICU length of stay, invasive ventilator-free days, escalation of oxygen modality (defined as change from baseline to addition of high flow oxygen, non-invasive ventilation, or continuous positive airway pressure), changes in oxygenation and respiratory rate as reported by the authors, and adverse events (as defined in the included trials).

### Data extraction and risk of bias assessment

Abstracted data included study characteristics (trial design, eligibility criteria, dates of recruitment, number of centers, countries); study population (age, sex, body mass index, severity of hypoxemia, and type of care unit (eg, ward or ICU) at enrolment); oxygenation modality at baseline; descriptions of trial intervention, control group, and co-interventions; and trial outcomes. Two authors, independently and in duplicate, assessed risk of bias using version 2 of the Cochrane risk-of-bias tool. Reviewers classified trials as low risk of bias, some concerns, or high risk of bias based on their assessment of five domains: bias arising from the randomization process, bias due to deviations from the intended intervention assignment, bias from missing outcome data, bias in measurement of the outcome, and bias in selection of the reported result.

### Data synthesis

The primary analysis was conducted using a frequentist approach. Dichotomous variables were pooled using a random effects model (DerSimonian and Laird), and effect estimates were reported as relative risks with corresponding 95% confidence intervals, and continuous variables as mean differences with corresponding 95% confidence intervals. Mean values and standard deviations were estimated from median and interquartile range when required, as previously described.^22^ Oxygen saturation to fraction of inspired oxygen (SpO_2_:FiO_2_) ratios were estimated from arterial oxygen tension to fraction of inspired oxygen (PaO_2_:FiO_2_) ratios as previously described.^23^ For cluster randomized controlled trials we planned to account for the design effect^17^ using intraclass correlation reported in the study or in other similar studies, but these trials did not contribute to any outcomes that were meta-analyzed. Trials with no events in both arms were excluded from primary analyses. We assessed the percentage of the total variance due to heterogeneity between trials using the I^2^ statistic.^24^ Intention-to-treat data were used whenever possible.

Preplanned secondary bayesian analyses for endotracheal intubation and mortality outcomes were also performed to assess the robustness of results according to varying and prespecified prior beliefs about the effect of awake prone positioning. The bayesian approach differs from the conventional frequentist approach. We used established informative priors for heterogeneity between studies.^25^ We used non-informative priors for mean effects, followed by those informed by a previously published meta-analysis of controlled observational studies involving 1526 patients pooled from 10 studies^9^ (see supplemental eTable 1 for intubation priors and mortality priors) and hypothetical ones based on a proposed framework in critical care.^25^ ^26^ Priors were defined and declared a priori. Bayesian random effects meta-analysis was performed using normal-normal hierarchical models and a hybrid random walk Metropolis-Hastings algorithm with Gibbs updates and blocked model parameters, four chains, random initial chain values, a minimum of 40 000 Markov chain Monte Carlo samples with 10 000 burn-in, and thinning of 10 to estimate posterior distributions of effects. Convergence was confirmed visually and with Gelman-Rubin diagnostic statistics all less than 1.1 (see supplemental eFigure 7). Results from the bayesian analyses were reported as relative risks and corresponding centile based 95% credible intervals.

Trial sequential analysis was performed to assess risks of random error in the conventional meta-analyses and if the required information size assumptions were met according to prespecified effect sizes of interest (see supplemental eMethods 3).^27^

In addition, we performed several preplanned subgroup analyses according to risk of bias, duration of awake prone positioning, severity of baseline hypoxemia, geographic/economic setting, location at randomization, and baseline mode of oxygen delivery. The cut points defining the subgroups for duration of awake prone positioning (≥5 h/day *v* <5 h/day and severity of baseline hypoxemia (SpO_2_:FiO_2_ <150 *v* ≥150) were chosen as they approximated the median values in the COVI-PRONE trial^14^ and the Ehrmann et al prospective meta-analysis,^11^ which represented the largest trials with data available to us at the time of protocol development. Our assumption was that these cut points would approximate the median of the medians across all trials. We conducted several preplanned sensitivity analyses: excluding unpublished trials (ie, abstracts and preprints), trials reported as stopping early, outcomes from the individual trials of the prospective meta-analysis (and instead substituting with pooled outcomes from the prospective meta-analysis of randomized trials), trials with no events in either arm, cluster randomized trials, quasi-randomized trials, and studies with more than low risk of bias. A post hoc sensitivity analysis was conducted with a random effects model using a restricted maximum likelihood approach with the Hartung-Knapp-Sidik-Jonkman confidence interval correction.^28^ Because more randomized controlled trials were identified than anticipated, we modified the analysis plan post hoc to exclude any quasi-randomized trials from the primary and secondary outcome analyses and instead include such trials in a sensitivity analysis. We performed a preplanned meta-regression to assess the association between the average daily duration of awake prone positioning (predictor variable) and the primary outcome of endotracheal intubation. We examined small study effects by inspecting funnel plots and the results of Egger’s test.^29^

Frequentist and bayesian analyses were performed in STATA (Stata version 16.0 and 17.0). We used trial sequential analysis software (version 0.9.5.10 Beta, Copenhagen Trial Unit, Center for Clinical Intervention Research, Rigshospitalet, Copenhagen, Denmark). Two sided P values <0.05 were considered statistically significant. GradePro software was used to summarize Grading of Recommendations, Assessment, Development, and Evaluation (GRADE) recommendations and to calculate absolute effect calculations based upon the baseline risk and relative effect size.

We used the GRADE approach to assess the certainty of evidence for every outcome based on the following domains: risk of bias, inconsistency, indirectness, imprecision, and publication bias.^30^ Certainty of the evidence was classified as high, moderate, low, or very low.

### Patient and public involvement

Two members of the public with experience of covid-19 were engaged about the systematic review and meta-analysis. They shared that awake prone positioning was important and any treatment that could reduce the likelihood of intubation was meaningful and important from a patient perspective. One patient partner associated with one of the centers reviewed the revised manuscript for feedback.

## Results

### Search results

**Fig 1:**
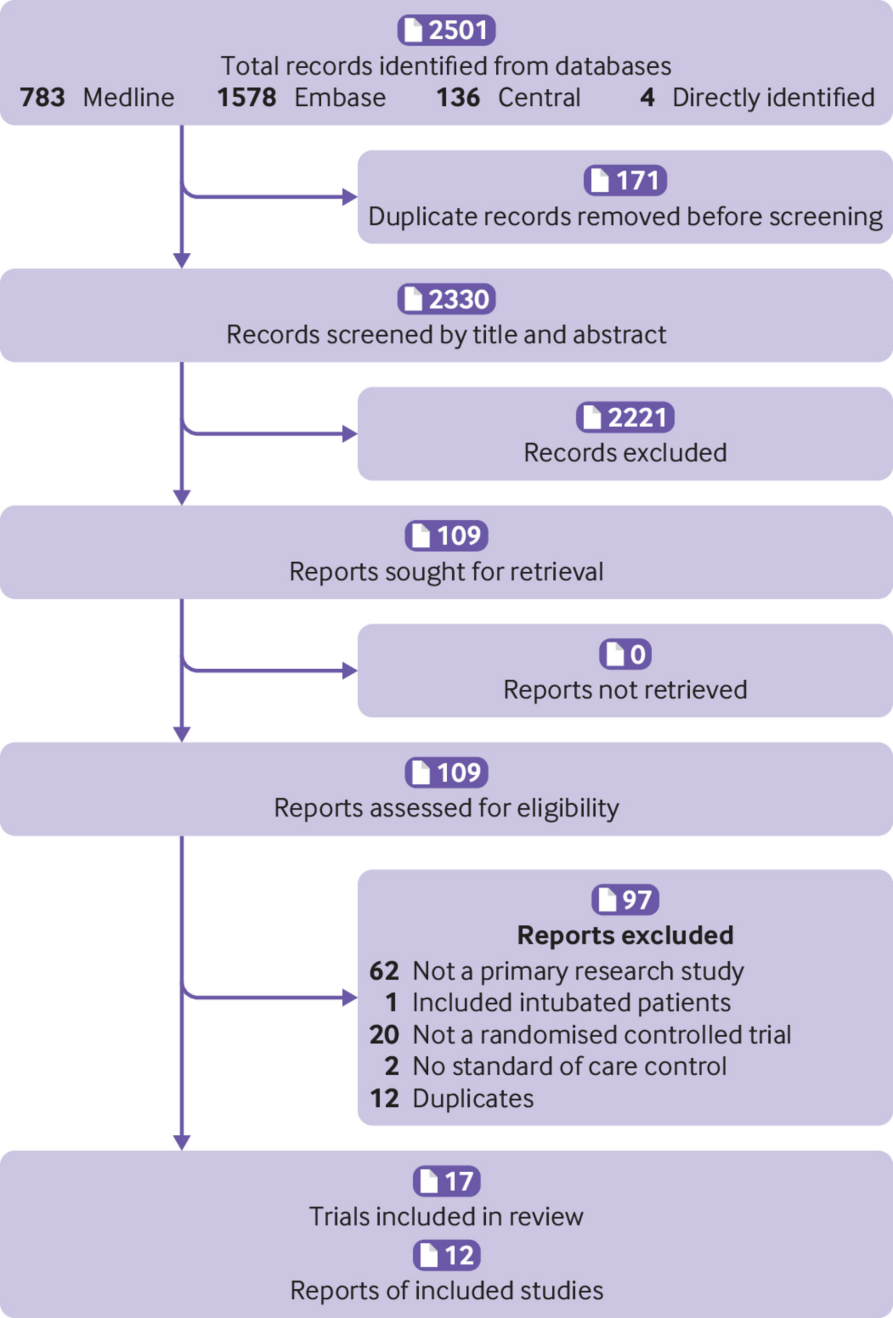
Summary of trial identification for review and meta-analysis. *Twelve articles representing 17 separate trials were identified. One article was a prospective meta-analysis of six individual randomized trials

Of 2330 citations, 109 articles underwent full text review (fig 1). Seventeen trials from 12 publications met the eligibility criteria and were included in the quantitative analysis.^11^ ^12^ ^14^ ^15^ ^16^ ^31^ ^32^ ^33^ ^34^ ^35^ ^36^ ^37^

### Trial and patient characteristics

The 17 included trials enrolled 2931 patients (table 1).^11^ ^12^ ^14^ ^15^ ^16^ ^31^ ^32^ ^33^ ^34^ ^35^ ^36^ ^37^ Six individual randomized controlled trials (1126 patients) were reported together in one publication as a prospective meta-analysis.^11^ We extracted data and outcomes from each individual trial separately whenever possible. Fourteen conventional randomized controlled trials enrolled 2363 patients,^11^ ^12^ ^14^ ^15^ ^31^ ^32^ ^33^ ^35^ two cluster randomized controlled trials enrolled 67 patients,^34^ ^36^ and one quasi-randomized trial enrolled 501 patients.^16^ Reviewers identified one unpublished trial that was included in a recent meta-analysis^12^ and three trials based on trial registrations identified in our search that were subsequently published.^14^ ^16^ ^37^

**Table 1:** Characteristics of included trials examining awake prone positioning in non-intubated adults with hypoxemic respiratory failure due to covid-19

Source; trial design	No of participants	Population	Location at enrolment	No (%) women	Median (IQR) baseline oxygenation		Prone positioning intervention	Control	Primary outcome	Follow-up duration	Median (IQR) duration of prone positioning (intervention group)
					Intervention	Control					
Alhazzani (Canada, USA, Kuwait, and Saudi Arabia) 2022; RCT	400	Suspected or confirmed covid-19. Requiring NP, HFNC, or NIV with a FiO_2_ ≥40%	HDU and ICU	117 (29)	S/F 132 (103-174)	S/F 136 (110-181)	8-10 hours/day	Usual care	Endotracheal intubation	30 days	5 (2-8) hours/day (≤4 days)
Ehrmann (Canada) 2021; MT-RCT	13	Confirmed covid-19. Requiring HFNC, P/F <300	Medical ward, HDU, and ICU*	6 (46)	S/F 169.3 (68.1)	S/F 166.8 (86.5)	As tolerated, and HFNC	HFNC+usual care	Composite: Endotracheal intubation or death	28 days	2.4 (1.7-3.0) hours/day (≤14 days)
Ehrmann (France) 2021; MT-RCT	402	Confirmed covid-19 Requiring HFNC and P/F <300	ICU	100 (25)	S/F 155.2 (48.3)	S/F 155.8 (44.6)	As tolerated, and HFNC	HFNC+usual care	Composite: Endotracheal intubation or death	28 days	2.0 (1.0-3.7) hours/day (≤14 days)
Ehrmann (Ireland) 2021; MT-RCT	24	Confirmed covid-19. Requiring HFNC or venturi mask with SpO_2_ <94%	Medical ward, HDU, and ICU*	8 (33)	S/F 193.9 (45.5)	S/F 178.3 (52.7)	As tolerated	Usual care	Composite: Endotracheal intubation or death	28 days	3.1 (2.1-3.9) hours/day (≤14 days)
Ehrmann (Mexico) 2021; MT-RCT	430	Confirmed covid-19. Requiring HFNC with FiO_2_ ≥30% to maintain SpO_2_ ≥90%	Medical ward, HDU, and ICU*	172 (40)	S/F 134.7 (38.7)	S/F 135.5 (37.9)	As tolerated, and HFNC	HFNC+usual care	Composite: Endotracheal intubation or death	28 days	8.6 (6.1-11.4) hours/day (≤14 days)
Ehrmann (Spain) 2021; MT-RCT	30	Confirmed covid-19. Requiring HFNC and P/F<300	ICU	7 (23)	S/F 162.9 (22.8)	S/F 155.8 (30.7)	As tolerated, and HFNC	HFNC+usual care	Composite: Endotracheal intubation or death	28 days	1.6 (1.1-2.3) hours/day (≤14 days)
Ehrmann (USA) 2021; MT-RCT	222	Confirmed covid-19. Requiring HFNC ≥50 L/min to maintain SpO_2_ 92-95% and P/F<200 or S/F<240	Medical ward, HDU, and ICU*	82 (37)	S/F 152 (37.8)	S/F 156 (40.6)	As tolerated and HFNC	HFNC+usual care	Composite: Endotracheal intubation or death	28 days	2.5 (0.7-6.9) hours/day (≤14 days)
Fralick (Canada and USA) 2021; RCT	248	Suspected covid-19 Requiring supplemental oxygen <50% FiO_2_	Medical ward	89 (36)	S/F 303 (261-336)	S/F 305 (267-339)	2 hours/session, 4 times/day, encouraged while sleeping	Usual care	Composite: In-hospital death, endotracheal intubation, NIV, or FiO_2_ ≥60% for 24 hours	30 days	6 (1.5-12.8) hours/72 hours (≤72 hours)
Rampon (USA and Spain) 2022; RCT	293	Suspected covid-19. Requiring <6 L/min supplemental oxygen	Medical ward	117 (40)	S/F 396 (308-457)	S/F 402 (311-457)	12 hours/day	Usual care	Composite: Respiratory deterioration (>2 L/min increase oxygen) or switch to different oxygen mode or ICU transfer	14 days	35.7% adhered to prone positioning >6 hours at least once
Harris (Qatar) 2022; RCT	61	Suspected covid-19. SpO_2_ <94% or supplemental oxygen >5 L/min	Medical ward	7 (11)	S/F 196 (165-245)	S/F 196 (182-240)	>3 hours to <16 hours/day	Usual care	Escalation of respiratory support	30 days	NR
Hashemian (Iran) 2021; RCT	75	Confirmed covid-19. Treated with NIV, P/F <300	ICU	23 (31)	Severity of P/F: mild, 233.1 (15.7); moderate, 138.4 (18.5); severe, 76.9 (13.0)	Severity of PF: mild, 213.4 (14.9); moderate, 150.7 (17.7); severe: 79.6 (13.3)	30 minutes every 4 hours, and NIV	Usual care and NIV	PaO_2_/FiO_2_	24 hours	NR
Jayakumar (India) 2021; RCT	60	Suspected covid-19. Requiring ≥4 L oxygen for SpO_2_ ≥92% or a P/F 100-300 and PaCO_2_ <45 mm Hg	ICU	10 (17)	P/F 233.2 (118.8)	P/F 185.6 (126.1)	6 hours/day	Usual care	Protocol adherence	7 days	Maximum session for prone positioning, 2 (1-3) hours (≤7 days)
Johnson (USA) 2021; RCT	30	Suspected covid-19. Admitted to hospital <48 hours	Medical ward	14 (47)	S/F NR. FiO_2_ 21% (21-29%)	S/F NR. FiO_2_ 21% (21-29%)	1-2 hours every 4 hours, or as tolerated	Usual care	Change in PaO_2_/FiO_2_	72 hours	1.6 (0.2-3.1) hours/72 hours
Kharat (Switzerland) 2021; C-RCT	27	Confirmed covid-19. With NP 1-6 L/min to maintain SpO_2_ 90-92%	Medical ward	10 (37)	S/F 318 (284-341)	S/F 336 (303-388)	12 hours/day	Usual care	Oxygen flow rate requirement	24 hours	4.9 (2.6) hours/day (≤24 hours)
Qian 2022 (USA); Q-RCT	501	Confirmed covid-19. Requiring supplemental oxygen for SpO_2_ ≥89%	Medical ward and ICU	217 (43)	S/F NR. Low flow oxygen (n=170)	S/F NR. Low flow oxygen (n=162)	As tolerated	Usual care	Highest level of oxygen support on the day 5 after enrollment (WHO COVID-19 Ordinal Outcome Scale)	5 days	4.2 (1.8-6.7) hours/day (≤5 days)
Rosén (Sweden) 2021; RCT	75	Confirmed covid-19. Requiring HFNC or NIV with a P/F ≤150 for >1 hour	Medical ward and ICU	20 (27)	S/F 151 (131-174)	S/F 157 (136-175)	16 hours/day	Usual care	Endotracheal intubation	30 days	9.0 (4.4-10.6) hours/day (≤72 hours)
Taylor 2021 (USA); C-RCT	40	Confirmed covid-19 with SpO_2_ <93% or requiring ≥3 L/min oxygen	Medical ward	13 (33)	S/F NR. NP <4 L/ min (n=15)	S/F NR. NP <4 L/ min (n=7)	As tolerated	Usual care	Implementation outcome framework	NR	No of participants attempting awake prone positioning, ≤48 hours (n=10)

ARDS=acute respiratory distress syndrome; C-RCT=cluster randomized controlled trial; FiO_2_=fraction of inspired oxygen; HDU=high dependency unit; HFNC=high flow nasal cannula; ICU=intensive care unit; IQR=interquartile range; MT-RCT=randomized controlled meta-trial; NIV=non-invasive ventilation; NP=nasal prongs; NR=not reported; PaO_2_=partial pressure of arterial oxygen; P/F=PaO_2_:FiO_2_ ratio; Q-RCT=quasi randomized controlled trial; S/F=SpO_2_:FiO_2_ ratio; RCT=randomized controlled trial; SpO_2_=oxygen saturation; WHO=World Health Organization.

* Location at enrollment not specified by trial site.

Supplemental eTable 2 presents the enrollment criteria for each trial. The median proportion of women in the awake prone positioning groups was 36% (interquartile range 25-40%) and in the usual care groups was 33% (23-40%). Median baseline peripheral oxygen saturation to fraction of inspired oxygen ratio (SpO_2_:FiO_2_) at randomization in the awake prone positioning groups was 169 (interquartile range 152-233) and in the usual care groups was 167 (156-220). Four trials (567 patients) were conducted exclusively in ICUs,^11^ ^15^ ^32^ six trials (699 patients) were conducted on medical wards,^12^ ^31^ ^33^ ^34^ ^36^ ^37^ and seven trials (1665 patients) were conducted in mixed settings, including ICUs, high dependency units, and medical wards.^11^ ^14^ ^16^ ^35^

Management of the control group was usual care in 11 trials (1759 patients),^11^ ^12^ ^14^ ^16^ ^31^ ^32^ ^33^ ^34^ ^35^ ^36^ ^37^ high flow nasal cannula (similar to the intervention group) plus usual care in five trials (1097 patients),^11^ and non-invasive ventilation (similar to the intervention group) plus usual care in one trial (75 patients).^15^ For the intervention group, the target duration of awake prone positioning ranged from as tolerated in eight trials^11^ ^16^ ^36^ to at least 16 hours each day in one trial.^35^ The actual duration of prone positioning was reported in 13 trials,^11^ ^14^ ^16^ ^31^ ^32^ ^33^ ^34^ ^35^ with a median of 2.8 (interquartile range 2.2-5) hours per day.

### Risk of bias in included studies

Supplemental eTable 3 shows the risk of bias assessment for the primary outcome of endotracheal intubation, and supplemental eTable 4 shows the secondary outcome of mortality. Twelve of the 17 trials were classified as low risk of bias (2204 patients),^11^ ^14^ ^31^ ^34^ ^35^ ^36^ ^37^ three trials had some concerns (151 patients),^12^ ^32^ ^33^ and two trials (576 patients)^15^ ^16^ were classified as high risk of bias owing to allocation sequence generation^16^ and selection of reported results.^15^

### Primary outcome: endotracheal intubation

**Fig 2:**
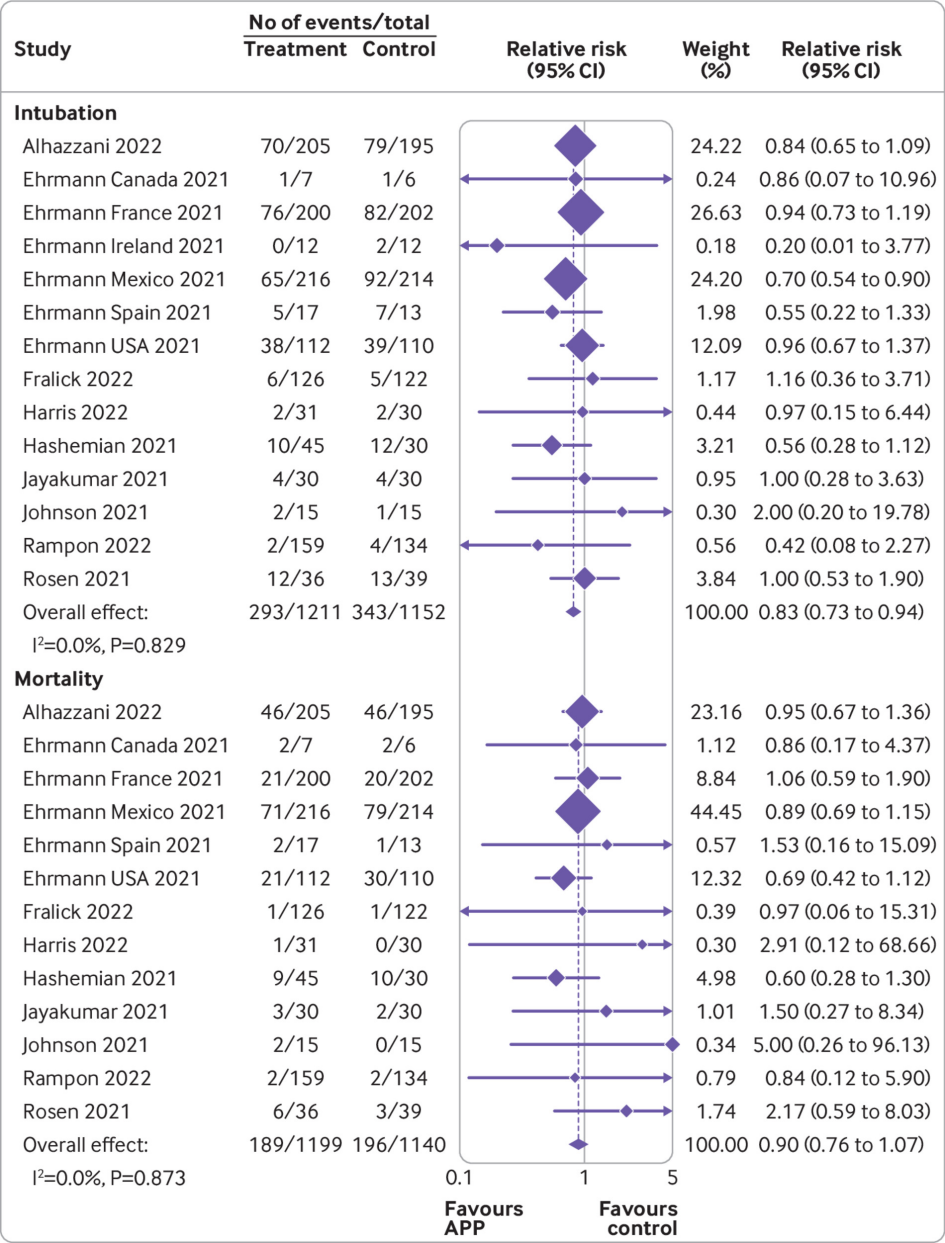
Forest plots for awake prone positioning compared with usual care for intubation and mortality in adults with hypoxemic respiratory failure due to covid-19. Six trials assessed intubation at 28 days (six Ehrmann trials), two trials assessed intubation at any time during hospital admission (Johnson, Fralick), three trials assessed intubation at 30 days (Alhazzani, Rosén, Harris), one trial assessed intubation at 14 days (Rampon), and two trials did not specify (Jayakumar, Hashemian). Two trials had no intubation events in both arms and were not included in this analysis (Taylor, Kharat). The quasi-randomized trial (Qian) was not included in this analysis. Six trials assessed mortality at 28 days (five Ehrmann trials, Harris), two trials assessed in-hospital mortality (Johnson, Fralick), two trials assessed mortality during intensive care unit admission (Jayakumar, Hashemian), one trial assessed mortality at 14 days (Rampon), one trial assessed mortality at 30 days (Rosén), and one trial assessed mortality at 60 days (Alhazzani). Three trials had no mortality events in both arms and were not included in this analysis (Ehrmann (Ireland), Taylor, Kharat). The quasi-randomized trial (Qian) was not included in this analysis. APP=awake prone positioning

Pooled analysis of 14 trials (2363 patients)^11^ ^12^ ^14^ ^15^ ^31^ ^32^ ^33^ ^35^ ^37^ for the primary outcome (fig 2) showed that awake prone positioning reduced the risk of endotracheal intubation compared with usual care (2363 patients; crude average 24.2% with awake prone positioning *v* 29.8% with usual care; relative risk 0.83 (95% confidence interval 0.73 to 0.94); I^2^=0%; high certainty). The absolute effect was 55 fewer intubations per 1000 patients (95% confidence interval 87 to 19 fewer intubations) receiving awake prone positioning. Visual inspection of the funnel plot and using Egger’s test suggested low risk of small study effects (see supplemental eFigure 1).

### Secondary outcomes

Pooled analysis of 13 trials (2339 patients)^11^ ^12^ ^14^ ^15^ ^31^ ^32^ ^33^ ^35^ ^37^ evaluating mortality (fig 2) did not show a significant difference in mortality between the two groups (2339 patients; 15.6% with awake prone positioning *v* 17.2% with usual care; 0.90 (0.76 to 1.07); I^2^=0%; high certainty). Visual inspection of the funnel plot and results of Egger’s test suggested a low risk of small study bias for mortality (see supplemental eFigure 2).

Three randomized trials (505 patients) reported ventilator-free days (see supplemental eFigure 3).^14^ ^33^ ^35^ The mean difference between awake prone positioning and usual care was 0.97 days (95% confidence interval −0.5 to 3.4); I^2^=9.8%; low certainty). Length of stay in the ICU (see supplemental eFigure 4) was reported in 11 randomized controlled trials (1792 patients).^11^ ^12^ ^14^ ^15^ ^32^ ^35^ No significant difference was found between awake prone positioning and usual care (−2.1 (−4.5 to 0.4); I^2^=86%; low certainty). Eleven randomized trials (1980 patients) reported on hospital length of stay (see supplemental eFigure 5).^11^ ^12^ ^14^ ^33^ ^35^ ^37^ Little to no difference was found between awake prone positioning and usual care (−0.09 days (−0.69 to 0.51); I^2^=0%; moderate certainty). Escalation of oxygen modality was reported in nine trials (1611 patients, see supplemental eFigure 6),^11^ ^14^ ^32^ ^33^ with no difference between the two groups (21.4% with awake prone positioning *v* 23.0% with usual care; relative risk 1.04 (95% confidence interval 0.74 to 1.44); I^2^=57%; low certainty).

The prospective meta-analysis of six trials and eight other trials reported on changes in oxygenation,^11^ ^14^ ^15^ ^16^ ^31^ ^32^ ^33^ ^34^ ^36^ and seven trials reported on changes in respiratory rate^11^ ^34^ (see supplemental eTable 5). Significant heterogeneity in the reported oxygenation indices and time of outcome assessment precluded pooling of data.

The most reported adverse events in the awake prone positioning groups (1469 patients) were unintentional dislodgement of vascular catheters (37 patients, 2.5%) and pain or discomfort (30 patients, 2%). Other reported adverse events in the awake prone positioning groups included nausea and vomiting (17 patients, 1.2%) and skin breakdown or pressure ulcers (10 patients, 0.7%) (see supplemental eTable 6).

### Bayesian analyses

The bayesian analysis using non-informative priors (table 2, supplemental eFigure 7) for endotracheal intubation showed a mean relative risk of 0.83 (95% credible interval 0.70 to 0.97: posterior probability for relative risk <0.95=96%). Similar results were found in analyses using informative priors (see supplemental eTable 1) that were enthusiastic, minimally skeptical, or moderately skeptical as well as hypothetical priors (table 2).

**Table 2:** Bayesian meta-analysis of endotracheal intubation and mortality outcomes

Priors*	Empiric priors						Hypothetical priors				
	Mean (95% CrI)	Pr(RR <1)	Pr(RR <0.95)	Pr(RR >1)	Pr(RR >1.05)		Mean (95%CrI)	Pr(RR <1)	Pr(RR <0.95)	Pr(RR >1)	Pr(RR >1.05)
**Intubation**											
Non-informative/neutral	0.83 (0.70 to 0.97)	0.99	0.96	0.01	0.00		0.84 (0.72 to 0.97)	0.99	0.95	0.01	0.00
Enthusiastic	0.76 (0.68 to 0.85)	1.00	1.00	0.00	0.00		0.82 (0.70 to 0.94)	1.00	0.98	0.00	0.00
Skeptical: minimal	0.82 (0.74 to 0.98)	1.00	1.00	0.00	0.00		0.83 (0.71 to 0.96)	0.99	0.97	0.01	0.00
Skeptical: moderate	0.89 (0.81 to 0.99)	0.96	0.90	0.01	0.00		0.84 (0.73 to 0.98)	0.99	0.95	0.01	0.00
**Mortality**											
Non-informative/neutral	0.90 (0.73 to 1.13)	0.83	0.68	0.17	0.08		0.91 (0.75 to 1.11)	0.83	0.68	0.17	0.08
Enthusiastic	0.83 (0.69 to 0.99)	0.98	0.93	0.02	0.01		0.87 (0.71 to 1.05)	0.93	0.82	0.07	0.03
Skeptical: minimal	0.91 (0.79 to 1.04)	0.87	0.73	0.09	0.02		0.92 (0.78 to 1.11)	0.81	0.62	0.19	0.08
Skeptical: moderate	0.96 (0.84 to 1.09)	0.68	0.47	0.25	0.09		0.96 (0.85 to 1.08)	0.74	0.44	0.26	0.08

Crl=credible interval; Pr(RR)=posterior probability of relative risk.

* Informative mean effect priors for the intubation outcome were: Enthusiastic 0.71 (95% CrI 0.61 to 0.82), skeptical: minimal 0.83 (0.72 to 0.95), skeptical: moderate 0.94 (0.82 to 1.07). Informative mean effect priors for the mortality outcome were: Enthusiastic 0.64 (95% CrI 0.44 to 0.92), skeptical: minimal 0.92 (0.76 to 1.10), skeptical: moderate 1.0 (0.84 to 1.20). See supplemental eTable 1 for expanded rationale and justification for mean effect priors. Informative between study variance priors were selected as previously described^25^ and include intubation (log normal distribution 3.93, 1.91^2^) and mortality (log normal distribution −4.17, 1.55^2^).

The bayesian analysis of mortality was concordant with the results of the frequentist analysis and suggested that the probability of benefit on mortality was relatively low, with a mean relative risk using a non-informative prior of 0.90 (95% credible interval 0.73 to 1.13: posterior probability for relative risk <0.95=68%, table 2). Table 2 presents estimates using the informative priors.

### Trial sequential analysis

Using trial sequential analysis, the relative risk for endotracheal intubation was 0.83 (trial sequential analysis adjusted confidence interval 0.70 to 0.99), which conclusively favored awake prone positioning (see supplemental eFigure 8). For mortality, the relative risk was 0.90 (0.45 to 1.82). The acquired information size was less than the required information size and no boundaries were crossed, therefore the trial sequential analysis was inconclusive for mortality (see supplemental eFigure 9). Similarly, the trial sequential analysis did not favor awake prone positioning for the other secondary outcomes, including ventilator-free days and ICU and hospital length of stay (see supplemental eFigure 9).

### Sensitivity analyses

Sensitivity analyses excluding one unpublished trial (354 patients)^12^ (see supplemental eFigure 10), two high risk of bias trials (576 patients),^15^ ^16^ and three trials with some concern for risk of bias (151 patients)^12^ ^32^ ^33^ (see supplemental eFigure 11) yielded results that were consistent with the primary analysis. Similarly, when excluding four trials (414 patients) that stopped early^12^ ^31^ ^33^ ^35^ (see supplemental eFigure 12), using overall pooled results from the prospective meta-analysis report^11^ (supplemental eFigure 13), excluding three trials (91 patients) with no events in either arm^11^ ^34^ ^36^ (see supplemental eFigure 14), and including one trial (501 patients) with quasi-randomized allocation^16^ (see supplemental eFigure 15), results were consistent with the primary analysis. A sensitivity analysis including one quasi-randomized trial^16^ did not change the posterior probabilities in the bayesian analysis for intubation and mortality. A post hoc sensitivity analysis was conducted with a random effects model using a restricted maximum likelihood approach with the Hartung-Knapp-Sidik-Jonkman confidence interval correction, which did not substantively change the results for endotracheal intubation and mortality outcomes (see supplemental eTable 7).

### Subgroup analyses

**Fig 3:**
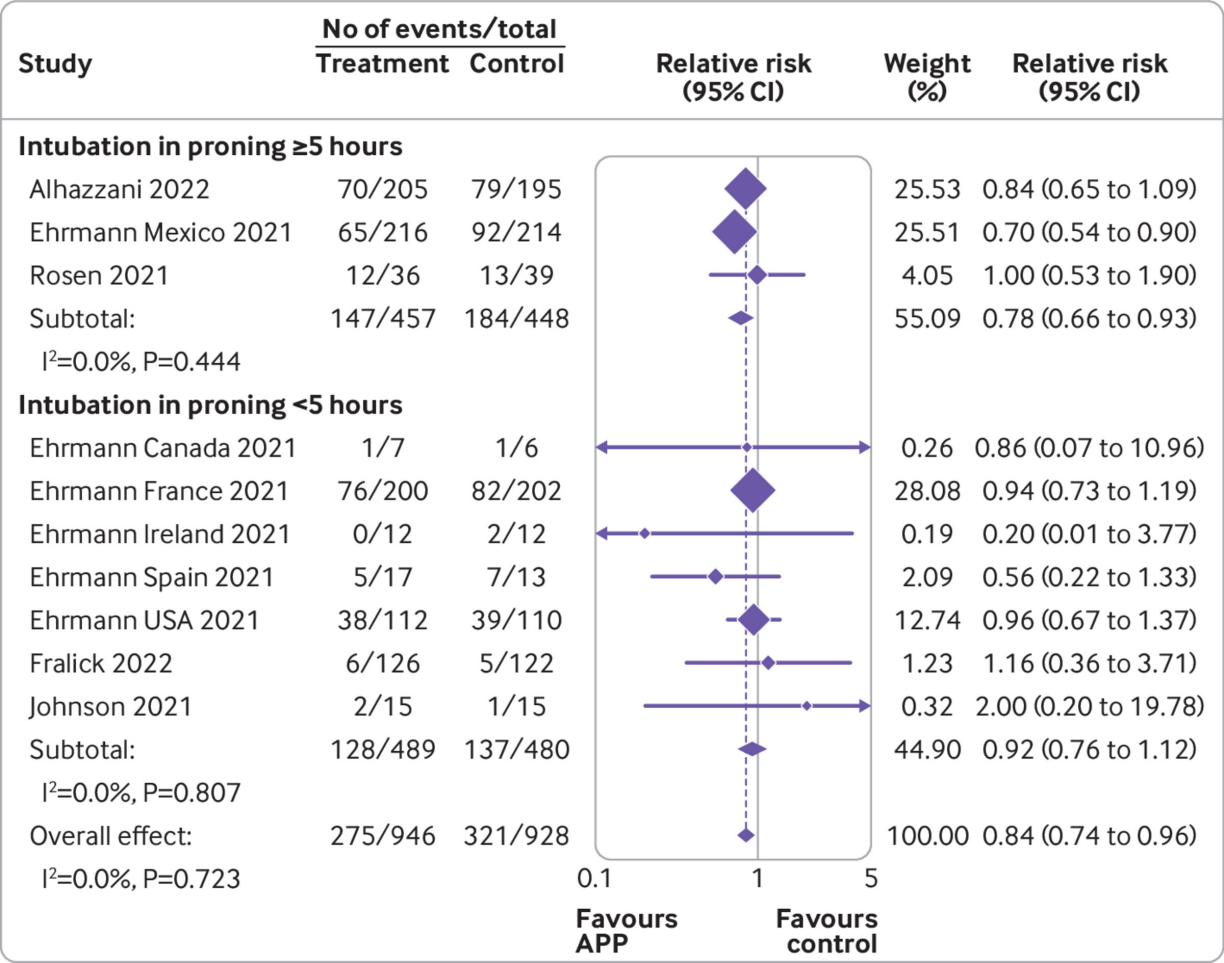
Forest plot for subgroup analysis of awake prone positioning compared with usual care for endotracheal intubation in patients with hypoxemic respiratory failure due to covid-19 according to duration of awake prone positioning. Two trials had no intubation events in both arms (Taylor, Kharat) and four trials that did not report the median duration of prone positioning (Jayakumar, Hashemian, Rampon, Harris) were excluded from this analysis. APP=awake prone positioning

**Fig 4:**
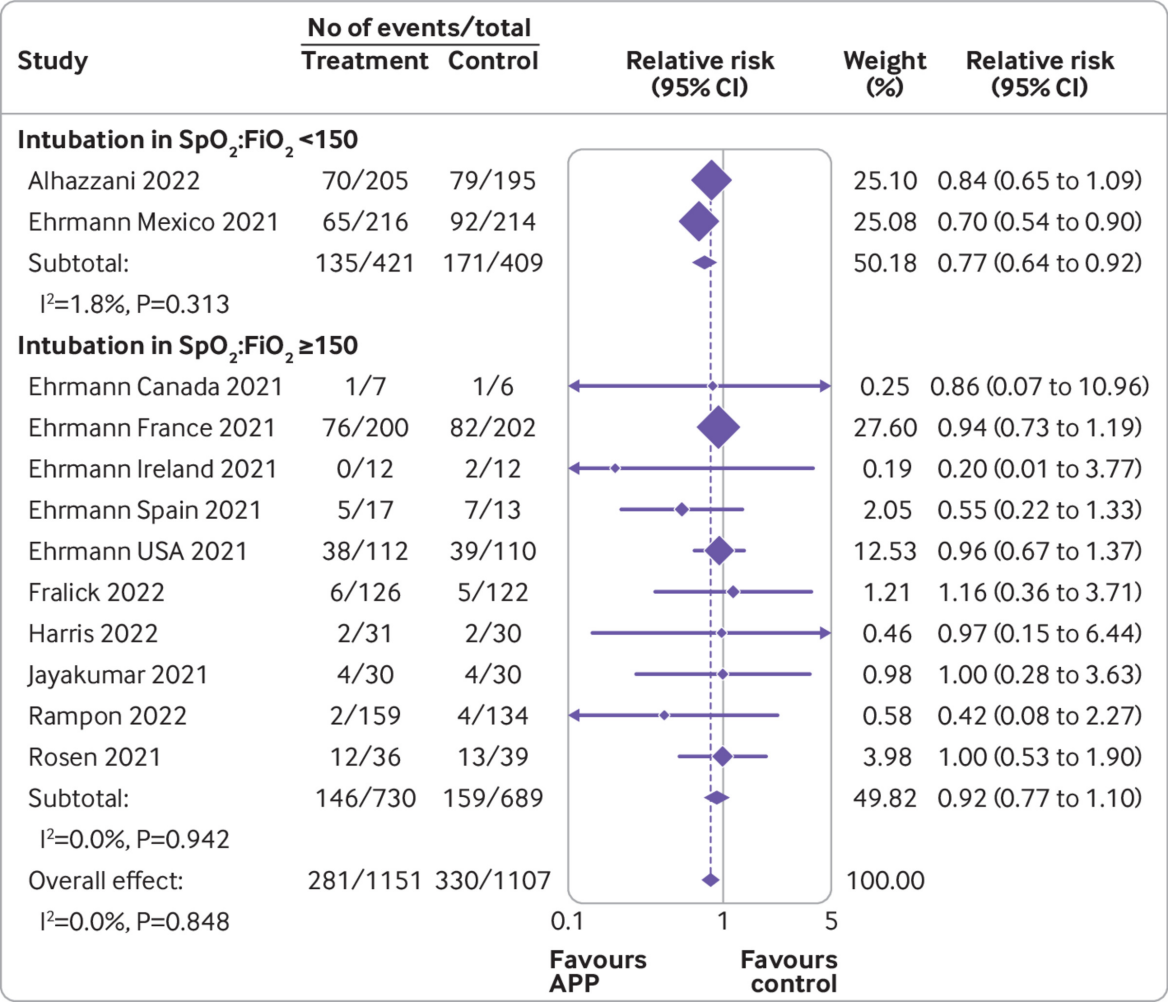
Forest plot for subgroup analysis of awake prone positioning compared with usual care for endotracheal intubation in patients with hypoxemic respiratory failure due to covid-19 according to median baseline oxygen saturation to fraction of inspired oxygen (SpO_2_:FiO_2_). Two trials had no intubation events in both arms (Taylor, Kharat) and three trials did not report the baseline SpO_2_:FiO_2_ (Johnson, Hashemian, Qian) and were excluded from this analysis. One trial reported baseline arterial oxygen tension to fraction of inspired oxygen (PaO_2_:FiO_2_), which was converted to SpO_2_:FiO_2_. APP=awake prone positioning

**Fig 5:**
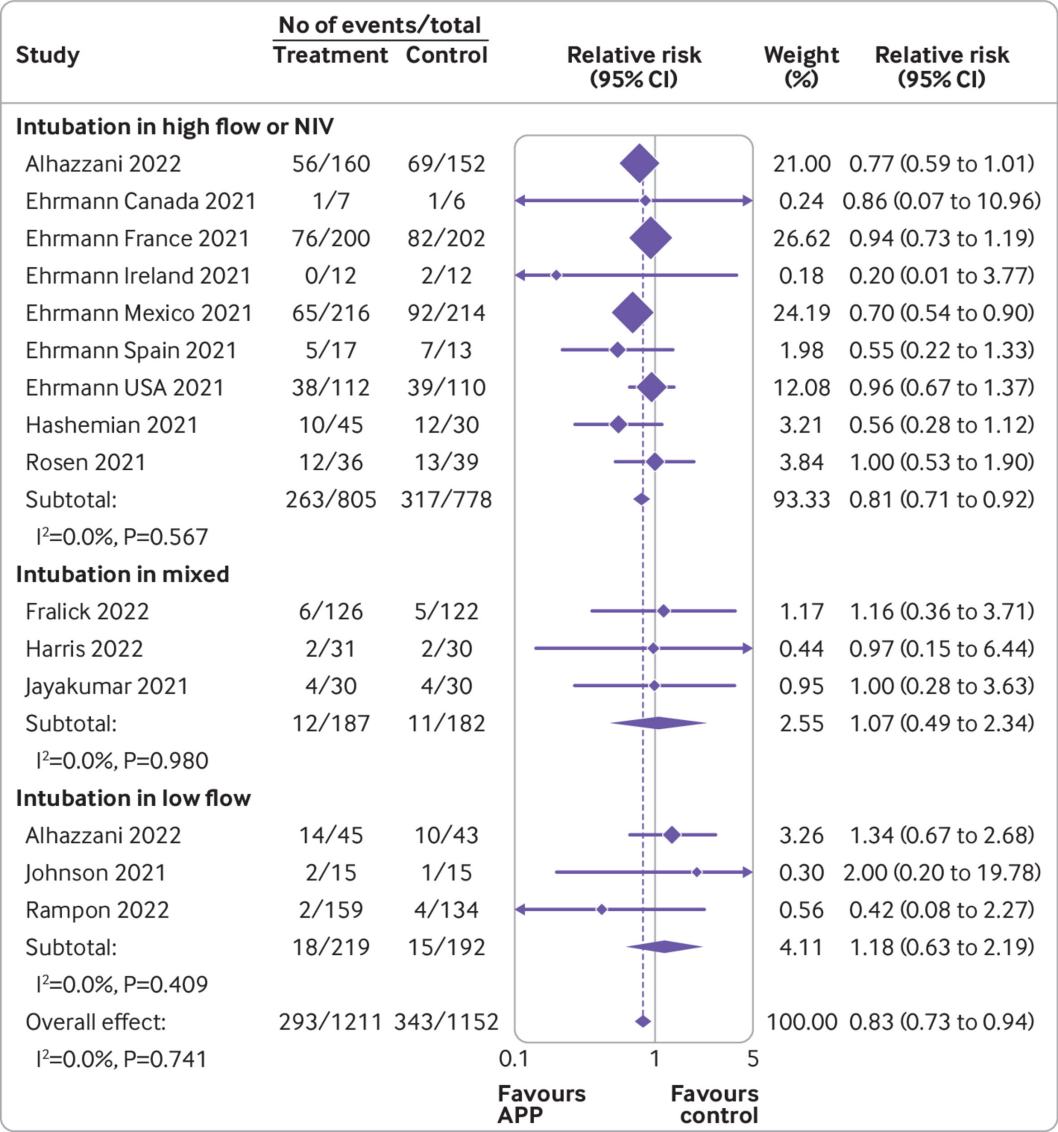
Forest plot for subgroup analysis of awake prone positioning compared with usual care for endotracheal intubation in patients with hypoxemic respiratory failure due to covid-19 according to baseline mode of oxygen delivery. Two trials had no intubation events in both arms (Taylor, Kharat) and were excluded from this analysis. One trial reported outcomes separately according to baseline mode of oxygen delivery (Alhazanni). APP=awake prone positioning; NIV=non-invasive ventilation

**Fig 6:**
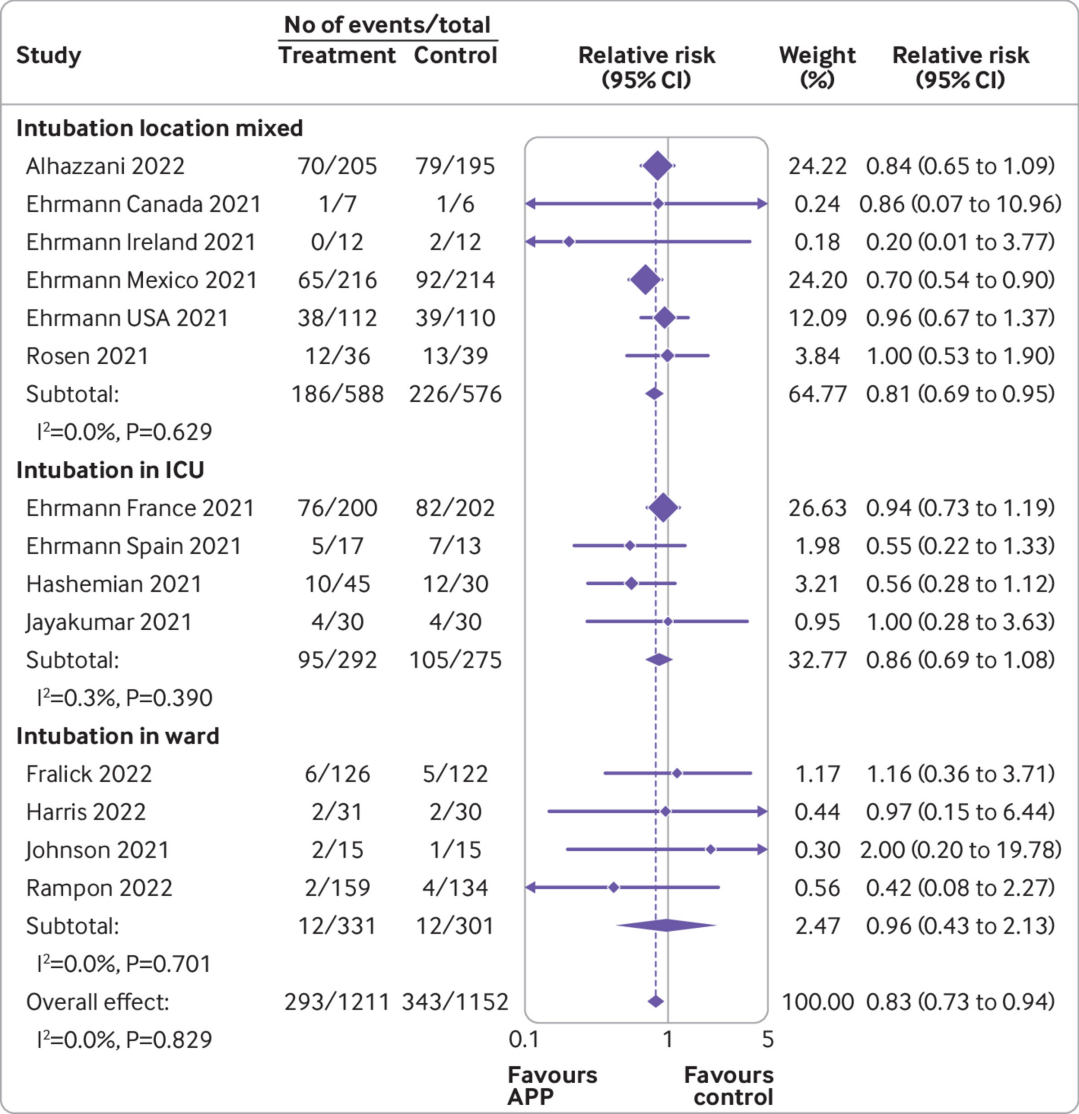
Forest plot for subgroup analysis of awake prone positioning compared with usual care for endotracheal intubation in patients with hypoxemic respiratory failure due to covid-19 according to location in hospital. Two trials had no intubation events in both arms (Taylor, Kharat) and were excluded from this analysis. APP=awake prone positioning; ICU=intensive care unit

**Fig 7:**
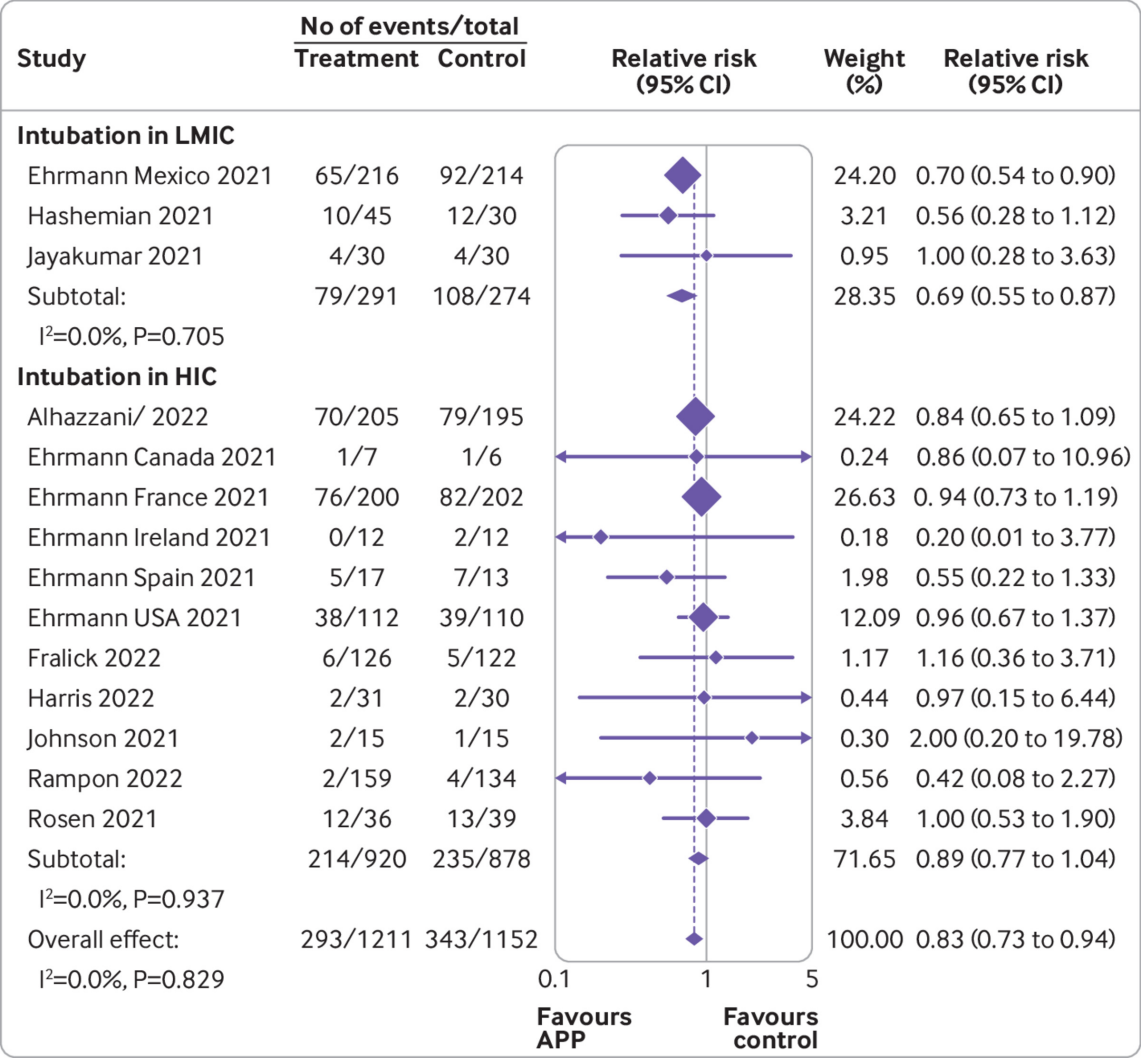
Forest plot for subgroup analysis of awake prone positioning compared with usual care for endotracheal intubation in patients with hypoxemic respiratory failure due to covid-19 according to country status (low or middle income and high income). Two trials had no intubation events in both arms (Taylor, Kharat) and were excluded from this analysis. Trials were classified as low or middle income countries or high income countries based on the Organisation for Economic Co-operation and Development in 2021. APP=awake prone positioning; HIC=high income countries; LMIC=low or middle income countries

Figure 3, figure 4, figure 5, figure 6, and figure 7 show the effect of awake prone positioning in prespecified subgroups for the primary outcome of endotracheal intubation. When trials were grouped according to trial level median duration of awake prone positioning, those with median duration of prone positioning ≥5 hours/day (three trials, 905 patients) showed a relative risk for endotracheal intubation of 0.78 (95% confidence interval 0.66 to 0.93; fig 3).^11^ ^14^ ^35^ In trials with a median duration of awake prone positioning <5 hours/day (seven trials, 969 patients)^11^ ^31^ ^33^ the relative risk was 0.92 (0.76 to 1.12, P for interaction=0.22). When trials were compared according to baseline severity of hypoxemia at trial level, the relative risk of endotracheal intubation in those with more severe hypoxemia (SpO_2_:FiO_2_ <150; two trials, 830 patients)^11^ ^14^ was 0.77 (0.64 to 0.92; fig 4), whereas in those trials with less severe baseline hypoxemia (SpO_2_:FiO_2_ ≥150; 10 trials, 1428 patients)^11^ ^12^ ^31^ ^33^ ^35^ ^37^ the relative risk was 0.92 (0.77 to 1.10, P for interaction=0.17). When the effect of awake prone positioning on endotracheal intubation was stratified by baseline oxygen mode of delivery, in trials exclusively using high flow oxygen or non-invasive ventilation at baseline (nine trials, 1583 patients)^11^ ^14^ ^15^ ^35^ the relative risk for endotracheal intubation was 0.81 (0.71 to 0.92; fig 5). In comparison, trials that used mixed modes of oxygen delivery (three trials, 369 patients)^12^ ^31^ ^32^ had a relative risk of 1.07 (0.49 to 2.34), and trials using only low flow oxygen (three trials, 411 patients)^14^ ^33^ ^37^ had a relative risk of 1.18 (0.63 to 2.19, P for interaction=0.81). One trial reported outcomes separately according to baseline mode of oxygen delivery and was pooled in two subgroups accordingly.^14^ When trials were stratified by type of hospital unit at randomization, those performed exclusively in ICUs (four trials, 567 patients)^11^ ^15^ ^32^ had a relative risk for endotracheal intubation of 0.86 (0.69 to 1.07) compared with 0.81 (0.69 to 0.95) in the six trials (1164 patients) performed in mixed settings (fig 6).^11^ ^14^ ^35^ In the four trials performed exclusively on general wards (632 patients),^12^ ^31^ ^33^ ^37^ the relative risk for endotracheal intubation was 0.96 (0.43 to 2.13, P for interaction=0.85). In 11 trials performed in high income countries (1798 patients),^11^ ^12^ ^14^ ^31^ ^33^ ^35^ ^37^ the relative risk for endotracheal intubation was 0.89 (0.77 to 1.04) compared with 0.69 (0.55 to 0.87, P for interaction=0.07) in three trials (565 patients)^11^ ^15^ ^32^ performed in low to middle income countries (fig 7).

When meta-regression was used, no significant association was found between the median daily duration of awake prone positioning and the log odds ratio for endotracheal intubation in 10 trials (1874 patients) that reported a mean or median duration of awake prone positioning (β coefficient −0.053, 95% confidence interval −0.14 to 0.03, P=0.19) (see supplemental eFigure 16).

### Certainty of evidence

Table 3 summarizes the details of the GRADE assessment of certainty of the evidence for the primary and secondary outcomes.

**Table 3:** Grading of Recommendations, Assessment, Development, and Evaluation (GRADE) in included randomized controlled trials

Outcomes	No of studies	Certainty assessment						No of patients		Effect		Certainty
		Risk of bias	Inconsistency	Indirectness	Imprecision	Other considerations		Awake prone positioning	No prone positioning	Relative (95% CI)	Absolute (95% CI)	
Intubation	13	Not serious*	Not serious†	Not serious	Not serious‡	None§		324/1211 (26.8%)	373/1152 (32.4%); 40.0% in the per protocol analysis	Relative risk 0.83 (0.73 to 0.94)	55 fewer (87 fewer to 19 fewer) per 1000; 68 fewer (108 fewer to 24 fewer) per 1000 in the per protocol analysis	High
Mortality	13	Not serious	Not serious	Not serious	Not serious	None**		243/1199 (20.3%)	243/1140 (21.3%)	Relative risk 0.90 (0.76 to 1.07)	21 (51 fewer to 15 more) per 1000	High
Ventilator-free days	4	Serious††	Not serious‡‡	Not serious	Serious§§	None¶¶^j^		2085	1997	-	Mean difference 0.53 days higher (0.19 lower to 1.24 higher)	Low
ICU length of stay	7	Not serious***	Serious†††	Not serious	Serious‡‡‡	None¶¶		2290	2190	-	Mean difference 1.78 days fewer (3.81 fewer to 0.24 more)	Low
Hospital length of stay	7	Not serious	Serious§§§	Not serious	Not serious	None¶¶		2290	2190	-	Mean difference 0.02 days more (0.93 fewer to 0.98 more)	Moderate
Escalation of oxygen modality	4	Not serious	Serious¶¶¶	Not serious	Serious****	None¶¶		174/814 (21.4%)	183/797 (23.0%)	Relative risk 1.04 (0.74 to 1.44)	9 more (from 60 fewer to 101 more) per 1000	Low

CI=confidence interval; ICU=intensive care unit.

* Although all 13 studies were unblinded and did not use a protocolized approach for endotracheal intubation, the certainly of the evidence was not rated down for risk of bias because endotracheal intubation is not a completely subjective outcome, and the nature of the intervention precludes blinding of healthcare workers. In addition, excluding studies at high risk of bias did not materially change the results.

† Visual inspection of the forest plot and statistical testing did not suggest heterogeneity between studies.

‡ Effect size is precise enough not to warrant rating down. There were >600 intubation events, the 95% CI is narrow, and the trial sequential analysis suggested the data are precise.

§ Inspection of funnel plot and results of Egger’s test (P=0.79) did not suggest publication bias.

¶ 40% endotracheal intubation rate in patients with covid-19 in the intensive care unit or acute care setting.

** Inspection of funnel plot and Egger’s test (P=0.25) did not suggest small study bias.

†† Rated down by one level for serious risk of bias. Two studies (Johnson et al and Qian et al) were judged to be at risk of selection bias. Qian et al contributed to 89% of the weight of the analysis.

‡‡ Although effect sizes varied between studies, the 95% CIs overlapped and statistical testing for heterogeneity was within acceptable limits.

§§ Rated down by one level for serious imprecision. The upper limit of the 95% CI included clinically important increase in ventilator-free days; however, the lower limit of the 95% CI included trivial reduction in ventilator-free days. Although this imprecision could have been a result of difference in study designs (risk of bias) there is uncertainty that this is the case.

¶¶ Unable to assess for publication bias given the small number of included studies.

*** Although four out of the three studies were judged to have some concerns or high risk for bias; subgroup analysis by risk of bias did not show a subgroup effect. Therefore, the certainty of evidence was not rated down for risk of bias.

††† Rated down by one level for serious heterogeneity. The forest plot showed variation in point estimates between studies (ranging from −5.8 days to 1.56 days) with some overlap in 95% CIs across studies. The I^2^ was 84% indicating significant heterogeneity. Only rated down by one level because the variation between studies was not implausibly large.

‡‡‡ Rated down by one level for serious imprecision. The 95% CI included large benefit (3.8 fewer days) and small harm (0.24 more days).

§§§ Rated down by one level for serious inconsistency. Although the I^2^ was 34.3%, the forest plot showed variability in point estimates between studies (range 3.4 fewer days to 17 days more in the hospital), there was some overlap in 95% CIs. The decision was made to rate down as this was the only category with concerns and the systematic review was to be conservative in assessment.

¶¶¶ Rated down by one level for serious inconsistency. The I^2^ was 57% suggesting some heterogeneity in treatment effects between included studies.

**** Rated down by one level for serious imprecision. The 95% CI included both significant reduction and increase in escalation of oxygen treatment.

## Discussion

### Principal findings

In this systematic review and meta-analysis of 17 trials, awake prone positioning was associated with a decreased risk of endotracheal intubation compared with usual care in adults with hypoxemic respiratory failure due to covid-19. The evidence of reduction in endotracheal intubation with awake prone positioning was of high certainty and the results were consistent across multiple sensitivity and bayesian analyses. On average, awake prone positioning resulted in 55 fewer intubations per 1000 patients (95% confidence interval 87 to 19 fewer intubations). However, awake prone positioning probably had little to no effect on mortality, ventilator-free days, ICU length of stay, hospital length of stay, escalation of oxygen treatment, or mode of oxygen delivery. Awake prone positioning is generally safe, with infrequent adverse events that include unintentional catheter dislodgement, discomfort, nausea, and skin breakdown.

### Comparison with other studies

As this systematic review represents a large number of patients and trials, the precision of the effect estimates is increased.^10^ ^12^ Including a larger number of trials addresses a limitation of previously published meta-analyses,^10^ ^12^ particularly by limiting any one trial from being excessively weighted in a meta-analysis. We also used two complementary statistical approaches (frequentist and bayesian) that supported the robustness of the results. The use of a bayesian approach allowed integration of prior information with our pooled data to determine a clinically useful summary of this information. Specifically, the bayesian approach provides probabilities of a benefit (or harm) with awake prone positioning given the observed data across varying previous beliefs (priors) about its effectiveness. For example, the posterior probability of a relative reduction of at least 5% in endotracheal intubation was high (≥0.90) across all degrees of prior beliefs about its effectiveness, given the data. In contrast, the posterior probability of a 5% relative reduction in mortality was 0.93 only if the prior beliefs about the effectiveness of awake prone positioning were strong (ie, using an enthusiastic prior). Many clinicians and patients would consider a 5% reduction in endotracheal intubation or mortality as clinically meaningful, particularly for a safe non-pharmacologic intervention.

The findings in this review were robust through a variety of different sensitivity analyses. The studies included in this systematic review differed from those in another recent meta-analysis,^12^ which included a randomized trial by Gad.^38^ We excluded that trial because it compared awake prone positioning without non-invasive ventilation with non-invasive ventilation, so the groups differed by the presence of prone positioning and by mode of respiratory support. In contrast, a trial by Hashemian and colleagues was incorporated in our review as it included non-invasive ventilation in both the usual care and the prone positioning groups.^15^ We a priori planned to include quasi-randomized trials in our analysis, anticipating a small number of eligible studies to be available for meta-analysis. One quasi-randomized trial was identified,^16^ in which allocation was based on patients’ medical record numbers, with even numbers receiving usual care and odd numbers receiving awake prone positioning. Owing to lack of concealed randomization, this study was assessed to be at high risk of selection bias. Although this quasi-randomized trial was not included in the primary analysis, when it was included in a sensitivity analysis the effect estimate did not change notably, further supporting the robustness of the results. The meta-analysis’s results are of important clinical relevance, as awake prone positioning is an inexpensive, non-pharmacological treatment that can be applied in a variety of hospital settings. In addition, awake prone positioning can be used in both low and middle income countries and high income countries, as shown by the geographic location of the studies in this systematic review.

Although we found no effect of awake prone positioning on mortality, a favorable effect cannot be excluded. Conversely, a reduction in the rate of endotracheal intubation was not associated with an increase in mortality, suggesting that patients were not put at risk by delaying intubation. To further support the safety of this intervention, the absolute rate of serious adverse events in the awake prone positioning group was low across trials. Also, downstream outcomes that could be associated with a reduction in endotracheal intubation, such as ventilator-free days and ICU and hospital length of stay were not statistically different between groups. Nevertheless, the effect estimates were consistently in the direction favoring awake prone positioning but with wide 95% confidence intervals. It may be that reducing intubation does not affect these outcomes, or that the lower number of studies reporting these secondary outcomes limited precision to detect small effect sizes.

The mechanism for how awake prone positioning reduces endotracheal intubation remains uncertain. Adherence to longer duration of prone positioning may be an effect modifier on the outcome of endotracheal intubation. It has been hypothesized that longer duration of awake prone positioning may be more effective, similar to placing patients in the prone position who are receiving invasive ventilation.^5^ ^13^ However, unlike patients receiving invasive ventilation who were placed in the prone positioning, awake patients are not sedated and not receiving neuromuscular blocking agents. This key difference may explain why none of the included trials that specified target durations for awake prone positioning met the prescribed dose in their intervention group. The intervention may be limited by patient tolerance as data suggest that awake patients may not cope well with long periods of prone positioning.^33^ Although many patients can place themselves in a prone position, others may need encouragement or assistance to do so for longer durations, which may require the availability of staff or other resources. Dedicated teams can increase adherence to prone positioning for intubated patients,^39^ ^40^ but data on the utility of this approach for non-intubated patients are limited. Other strategies to improve adherence, such as smart phone based guidance and reminders, did not result in better adherence in one trial.^37^ Thus, the benefits of awake prone positioning need to be weighed against the resources and staff needed to ensure safe adherence to the intervention. Thus, it remains uncertain whether better adherence to longer duration of awake prone positioning does modify the effect of the intervention. Our subgroup analysis suggested that in trials in which the median duration of awake prone positioning was ≥5 hours/day, the reduction in endotracheal intubation risk was relatively greater. However, the interaction test P value was not significant. Similarly, using meta-regression, the association between duration of awake prone positioning at the trial level and the effect size was not significant. Although these analyses suggest a potential association between duration of awake prone positioning and efficacy, they may be underpowered or potentially confounded since duration of prone positioning was not randomized and should be considered hypothesis generating. Even if an association exists between duration and efficacy, the optimal duration of awake prone positioning remains unknown. This question could be better evaluated in future randomized trials comparing various durations of prone positioning that are balanced with tolerability. In our other subgroup analyses, trials with more severe baseline hypoxemia, those performed in mixed hospital settings, and those performed in low to middle income countries tended to have larger effects. None of the interaction test P values were, however, significant, so we caution against over-interpretation of these findings. To most appropriately and efficiently allocate resources to deliver this intervention, future studies could aim to determine which patient subgroups, if any, benefit most from awake prone positioning.

### Strengths and limitations of this study

This meta-analysis should be interpreted within the context of its limitations. First, although we explored potential effect modification in subgroup analyses based on trial level characteristics, lack of individual patient data limited the ability to evaluate effect modification more precisely. For example, while many of the included trials overlapped the pre-vaccine and post-vaccine eras of the pandemic, it is unknown whether covid-19 vaccination status modifies the effectiveness of awake prone positioning. This could not be evaluated with the available data, but effect modifiers could be better studied using individual patient data meta-analysis. Second, owing to differences between the targeted and achieved duration of awake prone positioning across studies, we are unable to conclude whether there is an optimal duration of prone positioning for patients to benefit. Third, some of the planned analyses were limited because of heterogeneity in the definition and reporting of certain outcomes such as oxygenation, missing trial level data for some outcomes in the prospective meta-analysis,^11^ or because a few studies reported some outcomes, limiting precision and certainty. Fourth, the decision to intubate a patient can vary, with no fixed criteria. Furthermore, factors influencing the decision to intubate a patient were likely variable between providers and institutions and may have changed over the course of the pandemic. Despite this variability, the meta-analysis suggests there is high certainty in this finding based on the wide range of study locations (14 trials conducted in 12 different countries), and this finding is further supported by a secondary bayesian analysis and multiple sensitivity analyses. Finally, studies that are still in progress or were unpublished at the time this meta-analysis was completed might not be included and could influence the results. Although given the size and number of studies included in this review, such an influence would be unlikely unless the unpublished study was large, had a large treatment effect, or had multiple studies showing alternative effects to what we found. Strengths of this study include the adherence to quality standards for meta-analysis, use of GRADE to assess the certainty of evidence, and duplicate review of the search strategy and analysis for the primary outcome. This report includes a larger number of trials and patients than previous meta-analyses, uses rigorous sensitivity analyses to challenge the robustness of the primary analysis, and uses complementary preplanned bayesian analyses with a priori assumptions in addition to the traditional frequentist approach.

### Conclusions

Awake prone positioning compared with usual care reduced the risk of endotracheal intubation in adults with hypoxemic respiratory failure due to covid-19. Evidence on the effects of awake prone positioning on mortality or other secondary outcomes was, however, inconclusive. Adverse events related to awake prone positioning were uncommon, highlighting the safety of this intervention. However, adherence to the target duration of prone positioning was low in many trials. Thus, clinicians and patients must balance the goal of avoiding endotracheal intubation with the tolerability of awake prone positioning and availability of staff resources to encourage and assist patients. Future trials should aim to determine strategies to improve tolerability and adherence, assess the optimal duration of awake prone positioning, and determine the effect of awake prone positioning from other causes of hypoxemic respiratory failure.

**What is already known on this topic**

Awake prone positioning is an inexpensive, non-pharmacological treatment that can be applied readily and easily in a variety of hospital settings
The effect of awake prone positioning in patients with covid-19 related hypoxemic respiratory failure on endotracheal intubation and other outcomes remains uncertain

**What this study adds**

In this systematic review and meta-analysis of 17 randomized trials, awake prone positioning for hypoxemic respiratory failure due to covid-19 reduced the risk of endotracheal intubation, but evidence for the effect on mortality or other outcomes was inconclusive
Adverse events during awake prone positioning were uncommon and rarely serious

## Data Availability

No additional data available.

## Web extra

Extra material supplied by authors

## Notes

Contributors: JW and KKSP contributed equally and are joint first authors. All authors conceived and designed the study, analyzed and interpreted the data, and critically revised the manuscript. JW, KKSP, ZA, KL, KS, and WA acquired the data. JW, KKSP, and WA drafted the manuscript. JW, KKSP, DC, AG, NS, and WA performed the statistical analyses. JW, KP, and WA are guarantors of the data and manuscript. The corresponding author attests that all listed authors meet authorship criteria and that no others meeting the criteria have been omitted.

The lead authors (JW, KKSP, and WA) affirm that the manuscript is an honest, accurate, and transparent account of the study being reported; that no important aspects of the study have been omitted; and that any discrepancies from the study as originally planned (and, if relevant, registered) have been explained.

Dissemination to participants and related patient and public communities: We plan to engage the public and patients with lay summaries of the study results, disseminated through social media, conferences, and newsletters.

Provenance and peer review: Not commissioned; externally peer reviewed.

## References

[1] Lu R Zhao X Li J . Genomic characterisation and epidemiology of 2019 novel coronavirus: implications for virus origins and receptor binding. Lancet 2020;395:565-74. 10.1016/S0140-6736(20)30251-8. 32007145 PMC7159086

[2] Wu Z McGoogan JM . Characteristics of and Important Lessons From the Coronavirus Disease 2019 (COVID-19) Outbreak in China: Summary of a Report of 72 314 Cases From the Chinese Center for Disease Control and Prevention. JAMA 2020;323:1239-42. 10.1001/jama.2020.2648. 32091533

[3] Zhu N Zhang D Wang W China Novel Coronavirus Investigating and Research Team . A Novel Coronavirus from Patients with Pneumonia in China, 2019. N Engl J Med 2020;382:727-33. 10.1056/NEJMoa2001017. 31978945 PMC7092803

[4] Guérin C Reignier J Richard JC PROSEVA Study Group . Prone positioning in severe acute respiratory distress syndrome. N Engl J Med 2013;368:2159-68. 10.1056/NEJMoa1214103. 23688302

[5] Parhar KKS Zuege DJ Shariff K Knight G Bagshaw SM . Prone positioning for ARDS patients-tips for preparation and use during the COVID-19 pandemic. Can J Anaesth 2021;68:541-5. 10.1007/s12630-020-01885-0. 33367994 PMC7759020

[6] Munshi L Del Sorbo L Adhikari NKJ . Prone Position for Acute Respiratory Distress Syndrome. A Systematic Review and Meta-Analysis. Ann Am Thorac Soc 2017;14(Supplement_4):S280-8. 10.1513/AnnalsATS.201704-343OT. 29068269

[7] Weatherald J Solverson K Zuege DJ Loroff N Fiest KM Parhar KKS . Awake prone positioning for COVID-19 hypoxemic respiratory failure: A rapid review. J Crit Care 2021;61:63-70. 10.1016/j.jcrc.2020.08.018. 33096347 PMC7450241

[8] Solverson K Weatherald J Parhar KKS . Tolerability and safety of awake prone positioning COVID-19 patients with severe hypoxemic respiratory failure. Can J Anaesth 2021;68:64-70. 10.1007/s12630-020-01787-1. 32803468 PMC7427754

[9] Perez-Nieto OR Escarraman-Martinez D Guerrero-Gutierrez MA APRONOX Group . Awake prone positioning and oxygen therapy in patients with COVID-19: the APRONOX study. Eur Respir J 2022;59:2100265. 10.1183/13993003.00265-2021. 34266942 PMC8576803

[10] Fazzini B Page A Pearse R Puthucheary Z . Prone positioning for non-intubated spontaneously breathing patients with acute hypoxaemic respiratory failure: a systematic review and meta-analysis. Br J Anaesth 2022;128:352-62. 10.1016/j.bja.2021.09.031. 34774295 PMC8514681

[11] Ehrmann S Li J Ibarra-Estrada M Awake Prone Positioning Meta-Trial Group . Awake prone positioning for COVID-19 acute hypoxaemic respiratory failure: a randomised, controlled, multinational, open-label meta-trial. Lancet Respir Med 2021;9:1387-95. 10.1016/S2213-2600(21)00356-8. 34425070 PMC8378833

[12] Li J Luo J Pavlov I Awake Prone Positioning Meta-Analysis Group . Awake prone positioning for non-intubated patients with COVID-19-related acute hypoxaemic respiratory failure: a systematic review and meta-analysis. Lancet Respir Med 2022;10:573-83. 10.1016/S2213-2600(22)00043-1. 35305308 PMC8926412

[13] Weatherald J Norrie J Parhar KKS . Awake prone positioning in COVID-19: is tummy time ready for prime time? Lancet Respir Med 2021;9:1347-9. 10.1016/S2213-2600(21)00368-4. 34425072 PMC8378831

[14] Alhazzani W Parhar KKS Weatherald J COVI-PRONE Trial Investigators and the Saudi Critical Care Trials Group . Effect of Awake Prone Positioning on Endotracheal Intubation in Patients With COVID-19 and Acute Respiratory Failure: A Randomized Clinical Trial. JAMA 2022;327:2104-13. 10.1001/jama.2022.7993. 35569448 PMC9108999

[15] Hashemian SM Jamaati H Malekmohammad M Tabarsi P Khoundabi B Shafigh N . Efficacy of Early Prone Positioning Combined with Noninvasive Ventilation in COVID-19. Tanaffos 2021;20:82-5. 34976078 PMC8710224

[16] Qian ET Gatto CL Amusina O Vanderbilt Learning Healthcare System Platform Investigators . Assessment of Awake Prone Positioning in Hospitalized Adults With COVID-19: A Nonrandomized Controlled Trial. JAMA Intern Med 2022;182:612-21. 10.1001/jamainternmed.2022.1070. 35435937 PMC9016608

[17] Cochrane Handbook for Systematic Reviews of Interventions version 6.3 (updated Feb 2022): Cochrane; 2022. www.training.cochrane.org/handbook accessed March 1, 2022.

[18] Page MJ Moher D Bossuyt PM . PRISMA 2020 explanation and elaboration: updated guidance and exemplars for reporting systematic reviews. BMJ 2021;372:n160. 10.1136/bmj.n160. 33781993 PMC8005925

[19] Sampson M McGowan J Cogo E Grimshaw J Moher D Lefebvre C . An evidence-based practice guideline for the peer review of electronic search strategies. J Clin Epidemiol 2009;62:944-52. 10.1016/j.jclinepi.2008.10.012. 19230612

[20] Reeves BC Deeks JJ Higgins JPT , eds. Chapter 24. Including non-randomized studies on intervention effects. Cochrane, 2022, www.training.cochrane.org/handbook.

[21] Reeves BC Wells GA Waddington H . Quasi-experimental study designs series-paper 5: a checklist for classifying studies evaluating the effects on health interventions-a taxonomy without labels. J Clin Epidemiol 2017;89:30-42. 10.1016/j.jclinepi.2017.02.016. 28351692 PMC5669452

[22] Wan X Wang W Liu J Tong T . Estimating the sample mean and standard deviation from the sample size, median, range and/or interquartile range. BMC Med Res Methodol 2014;14:135. 10.1186/1471-2288-14-135. 25524443 PMC4383202

[23] Rice TW Wheeler AP Bernard GR Hayden DL Schoenfeld DA Ware LB National Institutes of Health, National Heart, Lung, and Blood Institute ARDS Network . Comparison of the SpO2/FIO2 ratio and the PaO2/FIO2 ratio in patients with acute lung injury or ARDS. Chest 2007;132:410-7. 10.1378/chest.07-0617. 17573487

[24] Guyatt GH Oxman AD Kunz R GRADE Working Group . GRADE guidelines: 7. Rating the quality of evidence--inconsistency. J Clin Epidemiol 2011;64:1294-302. 10.1016/j.jclinepi.2011.03.017. 21803546

[25] Turner RM Jackson D Wei Y Thompson SG Higgins JP . Predictive distributions for between-study heterogeneity and simple methods for their application in Bayesian meta-analysis. Stat Med 2015;34:984-98. 10.1002/sim.6381. 25475839 PMC4383649

[26] Zampieri FG Damiani LP Bakker J . Effects of a Resuscitation Strategy Targeting Peripheral Perfusion Status versus Serum Lactate Levels among Patients with Septic Shock. A Bayesian Reanalysis of the ANDROMEDA-SHOCK Trial. Am J Respir Crit Care Med 2020;201:423-9. 10.1164/rccm.201905-0968OC. 31574228

[27] Wetterslev J Jakobsen JC Gluud C . Trial Sequential Analysis in systematic reviews with meta-analysis. BMC Med Res Methodol 2017;17:39. 10.1186/s12874-017-0315-7. 28264661 PMC5397700

[28] Langan D Higgins JPT Jackson D . A comparison of heterogeneity variance estimators in simulated random-effects meta-analyses. Res Synth Methods 2019;10:83-98. 10.1002/jrsm.1316. 30067315

[29] Egger M Davey Smith G Schneider M Minder C . Bias in meta-analysis detected by a simple, graphical test. BMJ 1997;315:629-34. 10.1136/bmj.315.7109.629. 9310563 PMC2127453

[30] Guyatt GH Oxman AD Vist GE GRADE Working Group . GRADE: an emerging consensus on rating quality of evidence and strength of recommendations. BMJ 2008;336:924-6. 10.1136/bmj.39489.470347.AD. 18436948 PMC2335261

[31] Fralick M Colacci M Munshi L COVID Prone Study Investigators . Prone positioning of patients with moderate hypoxaemia due to covid-19: multicentre pragmatic randomised trial (COVID-PRONE). BMJ 2022;376:e068585. 10.1136/bmj-2021-068585. 35321918 PMC8941343

[32] Jayakumar D Ramachandran Dnb P Rabindrarajan Dnb E Vijayaraghavan Md BKT Ramakrishnan Ab N Venkataraman Ab R . Standard Care Versus Awake Prone Position in Adult Nonintubated Patients With Acute Hypoxemic Respiratory Failure Secondary to COVID-19 Infection-A Multicenter Feasibility Randomized Controlled Trial. J Intensive Care Med 2021;36:918-24. 10.1177/08850666211014480 33949237 PMC8107489

[33] Johnson SA Horton DJ Fuller MJ . Patient-directed Prone Positioning in Awake Patients with COVID-19 Requiring Hospitalization (PAPR). Ann Am Thorac Soc 2021;18:1424-6. 10.1513/AnnalsATS.202011-1466RL 33596394 PMC8513661

[34] Kharat A Dupuis-Lozeron E Cantero C . Self-proning in COVID-19 patients on low-flow oxygen therapy: a cluster randomised controlled trial. ERJ Open Res 2021;7:00692-2020. 10.1183/23120541.00692-2020 33718487 PMC7869594

[35] Rosén J von Oelreich E Fors D PROFLO Study Group . Awake prone positioning in patients with hypoxemic respiratory failure due to COVID-19: the PROFLO multicenter randomized clinical trial. Crit Care 2021;25:209. 10.1186/s13054-021-03602-9 34127046 PMC8200797

[36] Taylor SP Bundy H Smith WM Skavroneck S Taylor B Kowalkowski MA . Awake Prone Positioning Strategy for Nonintubated Hypoxic Patients with COVID-19: A Pilot Trial with Embedded Implementation Evaluation. Ann Am Thorac Soc 2021;18:1360-8. 10.1513/AnnalsATS.202009-1164OC 33356977 PMC8513648

[37] Rampon G Jia S Agrawal R . Smartphone-Guided Self-prone Positioning vs Usual Care in Nonintubated Hospital Ward Patients With COVID-19: A Pragmatic Randomized Clinical Trial. Chest 2022;162:782-91. 10.1016/j.chest.2022.05.009. 35597286 PMC9116967

[38] Gad GS . Awake prone positioning versus non invasive ventilation for COVID-19 patients with acute hypoxemic respiratory failure. Egypt J Anaesth 2021;37:85-90 10.1080/11101849.2021.1889944.

[39] Chiu M Goldberg A Moses S Scala P Fine C Ryan P . Developing and Implementing a Dedicated Prone Positioning Team for Mechanically Ventilated ARDS Patients During the COVID-19 Crisis. Jt Comm J Qual Patient Saf 2021;47:347-53. 10.1016/j.jcjq.2021.02.007. 33736966 PMC7907735

[40] Wells C Zhang Z Huelskamp S . Prone Team: A Large-Scale Prone Position Initiative During COVID-19 Pandemic. J Nurs Adm 2021;51:E13-7. 10.1097/NNA.0000000000001003. 33734184

