# Supplementary material for: Efficacy of awake prone positioning in patients with covid-19 related hypoxemic respiratory failure: systematic review and meta-analysis of randomized trials
Source: BMJ. 2022 Dec 7;379:e071966. doi: 10.1136/bmj-2022-071966 (PMC9727649; doi:10.1136/bmj-2022-071966)
Supplement: Supplementary file 1 — Supplementary information: Additional methods, tables, and figures [file weaj071966.ww.pdf]

Awake Prone Positioning for COVID-19 Hypoxemic Respiratory Failure: A Systematic Review and Meta-Analysis

Supplemental Data – Methods, Tables and Figures

EMETHODS 1 – PRISMA AND PRISMA- ABSTRACT CHECKLIST........ 3

EMETHODS 2 - SEARCH STRATEGY........ 8

EMETHODS 3 - TRIAL SEQUENTIAL ANALYSIS (TSA) METHODS........ 10

ETABLE 1 – SOURCES OF MEAN EFFECTS PRIORS FOR INTUBATION AND MORTALITY OUTCOMES FOR BAYESIAN ANALYSES........ 11

ETABLE 2 – DETAILED INCLUSION AND EXCLUSION CRITERIA FOR INCLUDED TRIALS........ 12

ETABLE 3 – RISK OF BIAS ASSESSMENT FOR INTUBATION........ 18

ETABLE 4 – RISK OF BIAS ASSESSMENT FOR MORTALITY........ 20

ETABLE 5 – SECONDARY OUTCOME – CHANGES IN OXYGENATION AND RESPIRATORY RATE WITH AWAKE PRONE POSITIONING........ 22

ETABLE 6 – REPORTED ADVERSE EVENTS........ 23

ETABLE 7 –SENSITIVITY ANALYSIS: COMPARISON OF RELATIVE RISK FOR PRIMARY OUTCOME OF INTUBATION AND SECONDARY OUTCOME OF MORTALITY USING RANDOM EFFECTS WITH THE DERSIMONIAN AND LAIRD APPROACH AND RANDOM EFFECTS WITH RESTRICTED MAXIMUM LIKELIHOOD WITH HARTUNG-KNAPP-SIDIK-JONKMAN CORRECTION........ 24

EFIGURE 1 – FUNNEL PLOT AND EGGER'S TEST FOR THE PRIMARY OUTCOME OF INTUBATION........ 25

EFIGURE 2 – FUNNEL PLOT AND EGGER'S TEST FOR SECONDARY OUTCOME OF MORTALITY........ 26

EFIGURE 3 – SECONDARY OUTCOME: VENTILATOR FREE DAYS........ 27

EFIGURE 4 – SECONDARY OUTCOME: INTENSIVE CARE UNIT LENGTH OF STAY........ 28

EFIGURE 5 - SECONDARY OUTCOME: HOSPITAL LENGTH OF STAY........ 29

EFIGURE 6 - SECONDARY OUTCOME: ESCALATION OF OXYGEN MODALITY........ 30

EFIGURE 7 – BAYESIAN TRACE, AUTOCORRELATION, HISTOGRAM, AND DENSITY PLOTS FOR MEAN EFFECT (THETA) AND BETWEEN-STUDY HETEROGENEITY (TAU2)........ 31

EFIGURE 9 – TRIAL SEQUENTIAL ANALYSIS FOR THE SECONDARY OUTCOMES........ 33

EFIGURE 10 – SENSITIVITY ANALYSIS FOR INTUBATION EXCLUDING UNPUBLISHED STUDIES............................ 36

EFIGURE 11 – SENSITIVITY ANALYSIS FOR INTUBATION EXCLUDING STUDIES AT RISK OF BIAS 37

EFIGURE 12 – SENSITIVITY ANALYSIS FOR INTUBATION EXCLUDING TRIALS THAT WERE STOPPED EARLY 38

EFIGURE 13 – SENSITIVITY ANALYSIS FOR ENDOTRACHEAL INTUBATION OUTCOME USING POOLED DATA FROM THE EHRMANN ET AL. PROSPECTIVE META-ANALYSIS.. 39

EFIGURE 14 – SENSITIVITY ANALYSIS FOR INTUBATION EXCLUDING TRIALS WITH NO EVENTS IN EITHER ARM .. 40

EFIGURE 15 –SENSITIVITY ANALYSES FOR INTUBATION AND MORTALITY OUTCOMES INCLUDING A TRIAL WITH QUASI-RANDOMIZED ALLOCATION.. 41

EFIGURE 16 – META-REGRESSION OF MEDIAN DURATION OF PRONE POSITIONING IN THE INTERVENTION GROUP AND INTUBATION... 43

eMethods 1 – PRISMA and PRISMA- abstract Checklist

PRISMA CHECKLIST

Section and Topic	Item #	Checklist item	Location where item is reported
**TITLE**			
Title	1	Identify the report as a systematic review.	1
**ABSTRACT**			
Abstract	2	See the PRISMA 2020 for Abstracts checklist.	6-7 (see completed PRISMA abstract checklist below)
**INTRODUCTION**			
Rationale	3	Describe the rationale for the review in the context of existing knowledge.	10
Objectives	4	Provide an explicit statement of the objective(s) or question(s) the review addresses.	10
**METHODS**			
Eligibility criteria	5	Specify the inclusion and exclusion criteria for the review and how studies were grouped for the syntheses.	11
Information sources	6	Specify all databases, registers, websites, organisations, reference lists and other sources searched or consulted to identify studies. Specify the date when each source was last searched or consulted.	11
Search strategy	7	Present the full search strategies for all databases, registers and websites, including any filters and limits used.	Supplement (eMethods-2)
Selection process	8	Specify the methods used to decide whether a study met the inclusion criteria of the review, including how many reviewers screened each record and each report retrieved, whether they worked independently, and if applicable, details of automation tools used in the process.	11-12
Data collection process	9	Specify the methods used to collect data from reports, including how many reviewers collected data from each report, whether they worked independently, any processes for obtaining or confirming data from study investigators, and if applicable, details of automation tools used in the process.	12
Data items	10a	List and define all outcomes for which data were sought. Specify whether all results that were compatible with each outcome	11

Section and Topic	Item #	Checklist item	Location where item is reported
		domain in each study were sought (e.g. for all measures, time points, analyses), and if not, the methods used to decide which results to collect.	
	10b	List and define all other variables for which data were sought (e.g. participant and intervention characteristics, funding sources). Describe any assumptions made about any missing or unclear information.	11-12
Study risk of bias assessment	11	Specify the methods used to assess risk of bias in the included studies, including details of the tool(s) used, how many reviewers assessed each study and whether they worked independently, and if applicable, details of automation tools used in the process.	11-12
Effect measures	12	Specify for each outcome the effect measure(s) (e.g. risk ratio, mean difference) used in the synthesis or presentation of results.	12-14
Synthesis methods	13a	Describe the processes used to decide which studies were eligible for each synthesis (e.g. tabulating the study intervention characteristics and comparing against the planned groups for each synthesis (item #5)).	12
	13b	Describe any methods required to prepare the data for presentation or synthesis, such as handling of missing summary statistics, or data conversions.	12-14
	13c	Describe any methods used to tabulate or visually display results of individual studies and syntheses.	12-14
	13d	Describe any methods used to synthesize results and provide a rationale for the choice(s). If meta-analysis was performed, describe the model(s), method(s) to identify the presence and extent of statistical heterogeneity, and software package(s) used.	12-13
	13e	Describe any methods used to explore possible causes of heterogeneity among study results (e.g. subgroup analysis, meta-regression).	12-14
	13f	Describe any sensitivity analyses conducted to assess robustness of the synthesized results.	13-14
Reporting bias assessment	14	Describe any methods used to assess risk of bias due to missing results in a synthesis (arising from reporting biases).	12
Certainty assessment	15	Describe any methods used to assess certainty (or confidence) in the body of evidence for an outcome.	14
RESULTS			
Study selection	16a	Describe the results of the search and selection process, from the number of records identified in the search to the number of	15 (figure 1)

Section and Topic	Item #	Checklist item	Location where item is reported
		studies included in the review, ideally using a flow diagram.	
	16b	Cite studies that might appear to meet the inclusion criteria, but which were excluded, and explain why they were excluded.	20
Study characteristics	17	Cite each included study and present its characteristics.	15 (table 1/eTable 2)
Risk of bias in studies	18	Present assessments of risk of bias for each included study.	15-16 (eTable 3 and eTable 4)
Results of individual studies	19	For all outcomes, present, for each study: (a) summary statistics for each group (where appropriate) and (b) an effect estimate and its precision (e.g. confidence/credible interval), ideally using structured tables or plots.	16-17 Fig 2/3
Results of syntheses	20a	For each synthesis, briefly summarise the characteristics and risk of bias among contributing studies.	eTable 3 and eTable 4
	20b	Present results of all statistical syntheses conducted. If meta-analysis was done, present for each the summary estimate and its precision (e.g. confidence/credible interval) and measures of statistical heterogeneity. If comparing groups, describe the direction of the effect.	16-19
	20c	Present results of all investigations of possible causes of heterogeneity among study results.	16-19
	20d	Present results of all sensitivity analyses conducted to assess the robustness of the synthesized results.	18-19 eFigure10-15
Reporting biases	21	Present assessments of risk of bias due to missing results (arising from reporting biases) for each synthesis assessed.	NA
Certainty of evidence	22	Present assessments of certainty (or confidence) in the body of evidence for each outcome assessed.	16-19, Table 3
**DISCUSSION**			
Discussion	23a	Provide a general interpretation of the results in the context of other evidence.	20
	23b	Discuss any limitations of the evidence included in the review.	20-23
	23c	Discuss any limitations of the review processes used.	20-23
	23d	Discuss implications of the results for practice, policy, and future research.	20-23
**OTHER INFORMATION**			
Registration and protocol	24a	Provide registration information for the review, including register name and registration number, or state that the review was not registered.	3, 11

Section and Topic	Item #	Checklist item	Location where item is reported
	24b	Indicate where the review protocol can be accessed, or state that a protocol was not prepared.	11
	24c	Describe and explain any amendments to information provided at registration or in the protocol.	11
Support	25	Describe sources of financial or non-financial support for the review, and the role of the funders or sponsors in the review.	2,7
Competing interests	26	Declare any competing interests of review authors.	2,3
Availability of data, code and other materials	27	Report which of the following are publicly available and where they can be found: template data collection forms; data extracted from included studies; data used for all analyses; analytic code; any other materials used in the review.	May be requested from the authors

PRISMA ABSTRACT CHECKLIST

Section and Topic	Item #	Checklist item	Reported (Yes/No)
TITLE			
Title	1	Identify the report as a systematic review.	Yes
BACKGROUND			
Objectives	2	Provide an explicit statement of the main objective(s) or question(s) the review addresses.	Yes
METHODS			
Eligibility criteria	3	Specify the inclusion and exclusion criteria for the review.	Yes
Information sources	4	Specify the information sources (e.g. databases, registers) used to identify studies and the date when each was last searched.	Yes
Risk of bias	5	Specify the methods used to assess risk of bias in the included studies.	Yes
Synthesis of results	6	Specify the methods used to present and synthesise results.	Yes
RESULTS			
Included studies	7	Give the total number of included studies and participants and summarise relevant characteristics of studies.	Yes
Synthesis of results	8	Present results for main outcomes, preferably indicating the number of included studies and participants for each. If meta-analysis was done, report the summary estimate and confidence/credible interval. If comparing groups, indicate the direction of the effect (i.e. which group is favoured).	Yes
DISCUSSION			
Limitations of evidence	9	Provide a brief summary of the limitations of the evidence included in the review (e.g. study risk of bias, inconsistency and imprecision).	Yes

Section and Topic	Item #	Checklist item	Reported (Yes/No)
Interpretation	10	Provide a general interpretation of the results and important implications.	Yes
OTHER			
Funding	11	Specify the primary source of funding for the review.	Yes
Registration	12	Provide the register name and registration number.	Yes

eMethods 2 - Search Strategy

Database: Ovid MEDLINE(R) and Epub Ahead of Print, In-Process, In-Data-Review & Other Non-Indexed Citations and Daily Search Strategy:

1	COVID-19/ or SARS-CoV-2/ or Coronavirus/ or Betacoronvirus/ or Coronavirus Infections/ (151888)
2	(covid* or coronavirus* or corona virus* or coronavirinae* or SARS-CoV* or SARSCoV* or SARS-corona* or nCoV* or n-CoV* or novel CoV* or 2019-nCoV* or hCoV* or h-CoV* or 2019-hCoV* or 2019hCoV* or "severe acute respiratory syndrome coronavirus 2" or "severe acute respiratory syndrome CoV 2").mp. (249201)
3	Prone Position/ (4679)
4	(proned or proning or self-pron*).mp. (280)
5	(prone adj5 position*).mp. (10195)
6	1 or 2 (249201)
7	3 or 4 or 5 (10323)
8	6 and 7 (783)

Database: Embase Search Strategy:

1	exp coronavirus disease 2019/ or exp severe acute respiratory syndrome coronavirus 2/ or coronavirinae/ or betacoronavirus/ or coronavirus infection/ (219243)
2	(covid* or coronavirus* or corona virus* or coronavirinae* or SARS-CoV* or SARSCoV* or SARS-corona* or nCoV* or n-CoV* or novel CoV* or 2019-nCoV* or hCoV* or h-CoV* or 2019-hCoV* or 2019hCoV* or "severe acute respiratory syndrome coronavirus 2" or "severe acute respiratory syndrome CoV 2").mp. (284410)
3	prone position/ (5118)
4	(proned or proning or self-pron*).mp. (652)
5	(prone adj5 position*).mp. (15182)
6	1 or 2 (284481)
7	3 or 4 or 5 (15529)
8	6 and 7 (1578)

Database: Central Search Strategy

#	Searches	Results
1	MeSH descriptor: [COVID-19] this term only	771
2	MeSH descriptor: [SARS-CoV-2] this term only	525
3	MeSH descriptor: [Coronavirus] this term only	4
4	MeSH descriptor: [Betacoronavirus] this term only	127
5	MeSH descriptor: [Coronavirus Infections] this term only	666
6	covid* or coronavirus* or corona virus* or coronavirinae* or SARS-CoV* or SARSCoV* or SARS-corona* or nCoV* or n-CoV* or novel CoV* or *nCoV or hCoV* or h-CoV* or *hCoV or "severe acute respiratory syndrome coronavirus 2" or "severe acute respiratory syndrome CoV 2"	11902

7	MeSH descriptor: [Prone Position] this term only	311
8	proned or proning or self-pron*	52
9	prone NEAR/5 position*	1809
10	{OR #1-#6}	11902
11	{OR #7-#9}	1825
12	#10 AND #11 in Trials	106
13	limit 12 to 25/09/2021 to 31/12/2021	13

eMethods 3 - Trial sequential analysis (TSA) methods

Trial sequential analyses (TSA) were conducted to assess risks of random error in the conventional meta-analyses and assess if the required information sizes (RIS) were met to accept or reject the proposed intervention effects (listed below). TSA settings corresponded to the conventional meta-analysis settings, i.e., DerSimonian-Laird random effects models with constant continuity correction. TSA were conducted using an alpha of 5% (overall 5% risk of type 1 error), a power of 90% (beta 10%), between-study heterogeneity according to the estimated diversity (D^2) statistic, and unweighted control event proportions from all included trials in the conventional meta-analyses (i.e., not considering trials with zero events in both arms) for binary outcomes, and we used the estimated variances for continuous outcomes. If the accrued information size is similar to or larger than the RIS, the TSA-adjusted CIs and conventional 95% CIs are be similar; if the accrued information size is less than the RIS, the TSA-adjusted CI will be appropriately widened according to an O-Brien-Fleming alpha-spending function, and Lan-DeMets TSA monitoring boundaries for benefit, harm and futility will be calculated. If the cumulative Z-score curve crosses any of the monitoring boundaries before the RIS is obtained, there is firm evidence for benefit/harm/futility.

The following pre-specified effect sizes of interest were used in the TSAs:

- Rate of intubation – relative risk reduction (RRR) of 15%
- Mortality – RRR of 10%

eTable 1 – Sources of mean effects priors for intubation and mortality outcomes for Bayesian analyses

**Intubation Mean Effects Priors**							
**Study / Prior**	**Justification**	**Proning group events**	**Proning group total**	**Control group Events**	**Control group Total**	**RR (95%CI)**	**OR (95%CI)**
Perez-Nieto 2021[1]	not used as a prior	77	311	123	311	0.63 (0.50 -0.80)	0.50 (0.36 -0.71)
Perez-Nieto 2021 Meta-analysis[1]	used to inform priors below	175	655	330	871	0.71 (0.61 -0.82)	0.60 (0.48 -0.75)
Enthusiastic	Based on Perez-Nieto 2021	175	655	330	871	0.71 (0.61 -0.82)	0.60 (0.48 -0.75)
Skeptical - minimal	Upper bound of the 95% CI of the enthusiastic estimate	205	655	330	871	0.83 (0.72 -0.95)	0.75 (0.60 -0.93)
Skeptical - moderate	Minimal Clinical Difference and includes the possibility of no effect	232	655	330	871	0.94 (0.82 -1.07)	0.90 (0.73 -1.11)
**Mortality Mean Effects Priors**							
**Study / Prior**	**Justification**	**Proning group events**	**Proning group total**	**Control Events**	**Control Total**	**RR (95% CI)**	**OR (95%CI)**
Perez-Nieto 2021[1]	not used as a prior	66	311	113	311	0.58 (0.45 -0.76)	0.47 (0.33 -0.67)
Perez-Nieto 2021 Meta-analysis[1]	used to inform priors below	105	649	206	849	0.64 (0.44 -0.92)	0.57 (0.35 -0.92)
Enthusiastic	Based on Perez-Nieto 2021	105	649	206	849	0.64 (0.44 -0.92)	0.57 (0.35 -0.92)
Skeptical - minimal	Upper bound of the 95% CI of the Enthusiastic estimate	145	649	206	849	0.92 (0.76 -1.10)	0.90 (0.70 -1.10)
Skeptical - moderate	Equal chance of benefit or harm with point estimate at no effect	158	649	206	849	1.00 (0.84 -1.20)	1.00 (0.79 - 1.27)

[1]Perez-Nieto OR, et al. Awake prone positioning and oxygen therapy in patients with COVID-19: the APRONOX study. Eur Respir J. 2022 Feb 24;59(2):2100265. doi: 10.1183/13993003.00265-2021.
RR: relative risk, CI: confidence interval, OR: odds ratio

eTable 2 – Detailed inclusion and exclusion criteria for included trials.

Study Author	Inclusion Criteria	Exclusion Criteria
**Alhazzani** 2022	1) Adults ≥ 18 years of age.<br>2) Suspected or confirmed Covid-19 (i.e., a positive polymerase chain reaction for SARS CoV-2 or pending polymerase chain reaction results).<br>3) Hypoxemia requiring oxygen supplementation ≥ 0.4 FiO2 or ≥ 5L nasal cannula.<br>4) Bilateral or unilateral chest infiltrates on x-ray as interpreted by the treating team.<br>5) Admitted to the ICU or an acute care unit where hemodynamic and respiratory monitoring is feasible	1) Immediate need for intubation as determined by the treating team.<br>2) Decreased level of consciousness (Glasgow Coma Scale score <10), or significant cognitive impairment hat may interfere with compliance (delirium, dementia)<br>3) Contraindication to proning including, but not limited to any of the following:<br>4) Open chest or abdomen, abdominal surgery (i.e., laparotomy) within the 4 days,<br>5) Unstable spine, facial, cervical, femur, or pelvic fractures.<br>6) Limited neck mobility or inability to lie prone comfortably<br>7) Skeletal deformities that interfere with proning.<br>8) Complete bowel obstruction.<br>9) Active upper gastrointestinal bleeding.<br>10) Patient is unlikely/unable to prone, or to be compliant as indicated by the treating team.<br>11) Body mass index > 40 kg/m^2<br>12) Pregnancy – third trimester.<br>13) Patient/substitute decision maker or caring physician's refusal to enroll in the study.<br>14) Patients with hemodynamic instability and moderate to high dose of vasopressors (norepinephrine dose ≥ 0.15 mcg/kg/min)<br>15) Intubation is not within patient`s goals of care<br>16) Patient received awake proning session for > 1 day prior to randomization<br>17) Previous intubation within the same hospital admission
**Ehrmann** (Canada) 2021	1) Covid-19 pneumonia based on the center for disease control guidelines<br>2) Presence of acute hypoxemic respiratory failure<br>3) Acute onset within 7 days of insult, or new (within 7 days) or worsening respiratory symptoms<br>4) Bilateral opacities on chest x-ray or computer tomographic scanner not fully explained by effusions, lobar or lung collapse, or nodules<br>5) Cardiac failure not the primary cause of acute respiratory failure	1) Patients with a consistent SpO2<80% when evaluated with a FiO2 of 0.6, or signs of respiratory fatigue (respiratory rate > 40/min, PaCO2>50 mmHg / pH 7.30, and obvious accessory respiratory muscle<br>2) Immediate need for intubation (PaO2/FiO2<50 mmHg or SpO2/FiO2 <90, unable to protect airway or mental status change)<br>3) Hemodynamic instability (sustained systolic blood pressure<br>4) Unable to collaborate with HFNC/proning with agitation or refusal of HFNC/proning

	6) Written informed consent 7) PaO2 / FiO2 ratio < 240 with HFNC at 50 L/min and SpO2 maintained at 92-95%	5) Chest trauma or any contraindication for proning<br>6) Pneumothorax<br>7) Age < 18 years<br>8) Pregnant<br>9) Body mass index > 40 kg/m2<br>10) Unable to communicate<br>11) Patient self-proned for more than 1 hr<br>12) Patient with moderate or severe ILD<br>13) Patient with stage IV lung cancer<br>14) Patient requiring long term oxygen therapy
**Ehrmann** (France)<br>2021	1) Adult patient suffering from Covid-19 pneumonia according to the diagnostic criteria in effect at the time of inclusion or very strongly suspected<br>2) Patient treated by nasal high flow therapy<br>3) Moderate or severe ARDS: bilateral radiological opacities not explained entirely by effusions, atelectasis or nodules; acute hypoxemia with worsening within the 7 previous days, not entirely explained by left ventricular failure; PaO2 / FiO2 ratio	1) Indication for immediate tracheal intubation<br>2) Significant acute progressive circulatory insufficiency<br>3) Impaired consciousness, confusion, restlessness<br>4) Body mass index> 40 kg / m2<br>5) Chest trauma or other contraindication to proning<br>6) Pneumothorax<br>7) Vulnerable person: safeguard of justice, curatorship or tutorship known at inclusion<br>8) Pregnant or lactating woman
**Ehrmann** (Ireland)<br>2021	1) Suspected or confirmed Covid-19 infection<br>2) Bilateral Infiltrates on chest X-ray SpO2	1) Age <18<br>2) Uncooperative or likely to be unable to lie on abdomen for 16 hours<br>3) Vomiting or bowel obstruction<br>4) Palliative care<br>5) Multiorgan failure<br>6)Standard contraindications to proning including the presence of an open abdominal wound, unstable pelvic fracture, spinal lesions and instability, pregnancy > 20/40 gestation and brain injury without monitoring of intracranial pressure.
**Ehrmann** (Mexico)<br>2021	1) Adult patients (18 y) with RT-PCR confirmed Covid-19 and respiratory distress (regardless of Berlin criteria for ARDS)<br>2) Requirement of a FiO2 ≥30% through high-flow nasal cannula (HFNC) to maintain a capillary SpO2 ≥90%<br>3) Written informed consent	1) Age<br>2) Pregnancy<br>3)Patients with immediate need of mechanical ventilation (altered mental status, signs of respiratory fatigue)<br>4) Any vasopressor requirement to maintain a median arterial pressure ≥65 mmHg<br>5) Contraindications for proning: recent abdominal or thoracic surgery/trauma, facial/pelvic/spine fractures, untreated pneumothorax)<br>6) Do not resuscitate or do not intubate order<br>7) Refusal or disability (uncooperative) of the patient to enroll in the study
**Ehrmann** (Spain)<br>2021	1) Adult patient suffering from Covid-19 pneumonia according to the	1) Indication for immediate tracheal intubation

	diagnostic criteria in effect at the time of inclusion or very strongly suspected<br>2) Patient treated by nasal high flow therapy<br>3) Moderate or severe ARDS: bilateral radiological opacities not explained entirely by effusions, atelectasis or nodules; acute hypoxemia with worsening within the 7 previous days, not entirely explained by left ventricular failure; PaO2 / FiO2 ratio	2) Significant acute progressive circulatory insufficiency<br>3) Impaired consciousness, confusion, restlessness<br>4) Body mass index> 40 kg / m2<br>5) Chest trauma or other contraindication to proning<br>6) Pneumothorax<br>7) Vulnerable person: safeguard of justice, curatorship or tutorship known at inclusion<br>8) Pregnant or lactating woman
**Ehrmann** (USA)<br>2021	1) Covid-19 pneumonia based on the center for disease control guidelines<br>2) Presence of acute hypoxemic respiratory failure<br>3) Acute onset within 7 days of insult, or new (within 7 days) or worsening respiratory symptoms<br>4) Bilateral opacities on chest x-ray or computer tomographic scanner not fully explained by effusions, lobar or lung collapse, or nodules<br>5) Cardiac failure not the primary cause of acute respiratory failure<br>6) Written informed consent 7) PaO2 / FiO2 ratio < 240 with HFNC at 50 L/min and SpO2 maintained at 92-95%	1) Patients with a consistent SpO2<80% when evaluated with a FiO2 of 0.6, or signs of respiratory fatigue (respiratory rate > 40/min, PaCO2>50 mmHg / pH 7.30, and obvious accessory respiratory muscle<br>2) Immediate need for intubation (PaO2/FiO2<50 mmHg or SpO2/FiO2 <90, unable to protect airway or mental status change)<br>3) Hemodynamic instability (sustained systolic blood pressure<br>4) Unable to collaborate with HFNC/proning with agitation or refusal of HFNC/proning<br>5) Chest trauma or any contraindication for proning<br>6) Pneumothorax<br>7) Age < 18 years<br>8) Pregnant<br>9) Body mass index > 40 kg/m2<br>10) Unable to communicate<br>11) Patient self-proned for more than 1 hr<br>12) Patient with moderate or severe ILD<br>13) Patient with stage IV lung cancer<br>14) Patient requiring long term oxygen therapy
**Fralick**<br>2021	1) Laboratory-confirmed or clinically highly suspected diagnosis of COVID-19, 2) Required supplemental oxygen (up to 50% FiO2)<br>3) Were able to independently adopt a prone position with verbal instruction	1) Prone positioning was contraindicated (e.g., recent abdominal surgery), impractical (e.g., dementia, severe delirium)<br>2) Mechanical intubation was indicated at the time of randomization as per the patient's treating physician
**Harris**<br>2022	1) Adults with suspected or confirmed COVID-19<br>2) SpO2 < 94% or supplement oxygen requirement of >5L/min<br>3) Requiring oxygen therapy in Hospital< 24 hours	1) Clinical assessment for immediate intervention.<br>2) PF<50<br>3) SF<90<br>4)RR>60bpm<br>5) Hemodynamics instability with need for vasopressors.<br>6) Multisystem organ failure<br>7) Age<18<br>8) Pregnancy<br>9) Impaired LOC, agitation or lack of cooperative patient.

		10) BMI>40<br>11) Unstable spine or pelvis<br>12) Abdominal wound<br>13) Pneumothorax<br>14) Any injury or illness that may be worsened or result in pain as a result of prone position.<br>15) Do not resuscitate order<br>16) Any contraindication to prone position
**Hashemia**<br>2021	1) COVID-19 patients<br>2) Admitted to ICU<br>3) Presence of ARDS defined by Berlin criteria	1) BMI < 18 or >30<br>2) Requiring emergent intubation<br>3) Already intubated
**Jayakumar**<br>2021	1) >18 years<br>2) Requiring 4 or more liters per minute of supplemental oxygen to maintain SpO2>92%or if ABG was available, PaO2/FiO2 ratio between 100 and 300 mmHg and PaCO2 < than 45mmHg	1) <18 years of age<br>2) Pregnant women<br>3) patients<br>with hemodynamic shock requiring norepinephrine >=0.1 mcg/<br>kg/min<br>4) GCS <15<br>5) Patients who needed immediate intubation in the opinion of the treating clinician<br>6) Absolute or relative contraindications to prone positioning (spinal instability secondary to severe rheumatoid arthritis, life threatening cardiac arrhythmias)
**Johnson**<br>2021	1) Symptoms of COVID-19 combined with either a high clinical suspicion and a pending COVID-19 assay or a positive COVID-19 assay within 10 days	1) Unable to change position without assistance<br>2) Pregnant,<br>3) Incarcerated<br>4) Admitted to an intensive care unit (ICU) or transfer was imminent<br>5) Mechanically ventilated<br>6) Receiving hospice
**Kharat**<br>2021	1) ≥18 years<br>2) Admitted to a medical ward for treatment of COVID-19 pneumonia<br>3) Low-flow oxygen therapy (defined as 1–6 L·min−1) through nasal cannulas to obtain a SpO2 level of 90–92%	1) Patients initially treated in the ICU or high-dependency unit and recovering from ARDS<br>2) those with oxygen needs >6 L·min−1 using a nasal cannula or with >40% inspiratory oxygen fraction (FiO2) using a Venturi mask to obtain a SpO2 level of 90–92%<br>3) pregnant women<br>4) terminally ill patients<br>5) and those unable to self-prone
**Qian**<br>2022	1) COVID-19 positive patients<br>2) Require supplemental oxygen	1) Patients admitted on mechanical ventilation will be excluded from enrollment.
**Rampon**<br>2022	Adult patient (> 18 years old)<br>(1) Admission to the medical wards or planned admission to the medical wards from the ED within the previous 48 hours (not ICU)	Baseline patient factors<br>(1) Inability to operate the hospital bed<br>(2) Inability to lie flat comfortably

	(2) confirmed COVID-19 infection or under evaluation for COVID-19<br>(3) Have access to their own functioning smartphone that can connect to the internet and receive text messages in the hospital room<br>(4) English or Spanish-speaking<br>(5) Ability to read simple instructions and answer simple written questions	(3) Inability to lie flat without shortness of breath -Inability to turn over independently<br>(4) Medical comorbidities<br>(5) Hemoptysis in the last 2 days<br>(6) Prior lung transplant<br>(7) Dementia<br>Acute issues<br>(1) Deep venous thrombosis treated for less than 2 days<br>(2) Unstable spine, femur, or pelvic fractures<br>(3) Mean arterial pressure lower than 65 mmHg<br>(4) Receiving ≥6 liters per minute of supplemental oxygen via nasal cannula, nasal pendant, or shovel mask<br>(5) Receiving supplemental oxygen via Venturi mask or non-rebreather mask<br>Recent interventions<br>(1) Chest tube in place<br>(2) Tracheal surgery or sternotomy during the previous 15 days -Serious facial trauma or facial surgery during the previous 15 days -Cardiac pacemaker inserted in the last 2 days<br>Other<br>(1) Known pregnancy or positive pregnancy test<br>(2) No negative pregnancy test in woman <50 years old<br>(3) Comfort measures only status<br>(4) Prisoner status<br>Previous enrollment in the APPEX-19 study or screened out/decided no
**Rosén**<br>2021	1) Adults (≥ 18 years old)<br>2) COVID-19 verified by positive SARS-CoV-2 reverse transcription polymerase chain reaction tests on naso- or oropharyngeal swabs<br>3) Hypoxemic respiratory failure, HFNO or NIV for respiratory support and a PaO2/ FiO2-ratio ≤ 20 kPa or corresponding values of SpO2 and FiO2 for more than one hour	1) Oxygen supplementation with a device other than HFNO or NIV<br>2) inability to assume prone or semi-prone position<br>3) immediate need for endotracheal intubation<br>4) severe hemodynamic instability<br>5) previous intubation for COVID-19 pneumonia<br>6) pregnancy<br>7) terminal illness with less than one year life expectancy<br>8) do-not-intubate order<br>9) inability to understand oral or written study information
**Taylor**<br>2021	1) Adult patients<br>2) Admitted to the hospital by one of the study teams<br>3) Tested positive for SARS-CoV-2 within 7 days or were suspected to have COVID-19 pneumonia	1) Patients contraindicated for APPS intervention (e.g., unable to self-turn)<br>2) Spinal instability, facial or pelvic fractures, open chest or abdomen<br>3) Altered mental status<br>4) Anticipated difficult airway<br>5) Signs of respiratory fatigue

	4) Experienced room air oxygen saturation < 93% or oxygen requirement of 3 L per minute or greater without the need for mechanical ventilation	6) Receiving end-of-life care

eTable 3 – Risk of bias assessment for intubation

Name (year)	Industry Sponsored	Randomization process	Assignment to intervention	Missing outcome data	Measurement of outcome	Selection of reported results	Other bias	Overall risk of bias
Alhazzani (2022)	No	LOW	LOW	LOW	LOW	LOW	Sample size was adjusted due to lower rate of intubation than anticipated	LOW
Ehrmann – Mexico	No	LOW	LOW	LOW	LOW	LOW	Study was terminated early due to meeting pre-defined criteria for efficacy.	LOW
Ehrmann – USA	No	LOW	LOW	LOW	LOW	LOW	Study was terminated early due to meeting pre-defined criteria for efficacy.	LOW
Ehrmann – Spain	No	LOW	LOW	LOW	LOW	LOW	Study was terminated early due to meeting pre-defined criteria for efficacy.	LOW
Ehrmann – France	No	LOW	LOW	LOW	LOW	LOW	Study was terminated early due to meeting pre-defined criteria for efficacy.	LOW
Ehrmann – Canada	No	LOW	LOW	LOW	LOW	LOW	Study was terminated early due to meeting pre-defined criteria for efficacy.	LOW
Ehrmann – Ireland	No	LOW	LOW	LOW	LOW	LOW	Study was terminated early due to meeting pre-defined criteria for efficacy.	LOW
Fralick (2022)	No	LOW	LOW	LOW	LOW	LOW	Stopped early for futility	LOW
Rampon (2022)	No	LOW	LOW	LOW	LOW	LOW	Unclear whether stopped early for futility or poor protocol adherence	LOW
Harris (2022)	No	SOME CONCERNS	SOME CONCERNS	LOW	LOW	LOW	Unclear whether stopped early or reported results at interim analysis	SOME CONCERNS
Hashemian (2021)	No	LOW	SOME CONCERNS	LOW	LOW	HIGH		HIGH
Jayakumar (2021)	No	LOW	SOME CONCERNS	LOW	LOW	LOW		SOME CONCERNS

Johnson (2021)	No	SOME CONCERNS	LOW	LOW	LOW	LOW	Stopped early for poor protocol adherence	SOME CONCERNS
Qian (2022)	No	HIGH	LOW	LOW	LOW	LOW		HIGH
Rosén (2021)	No	LOW	LOW	LOW	LOW	LOW	Stopped early for futility	LOW

eTable 4 – Risk of bias assessment for mortality

Name (year)	Industry Sponsored	Randomiza-tion process	Assignment to intervention	Missing outcome data	Measurement of outcome	Selection of reported results	Other bias	Overall risk of bias
Alhazzani (2022)	No	LOW	LOW	LOW	LOW	LOW	Sample size was adjusted due to lower rate of intubation than anticipated	LOW
Ehrmann – Mexico	No	LOW	LOW	LOW	LOW	LOW	Study was terminated early due to meeting pre-defined criteria for efficacy.	LOW
Ehrmann – USA	No	LOW	LOW	LOW	LOW	LOW	Study was terminated early due to meeting pre-defined criteria for efficacy.	LOW
Ehrmann – Spain	No	LOW	LOW	LOW	LOW	LOW	Study was terminated early due to meeting pre-defined criteria for efficacy.	LOW
Ehrmann – France	No	LOW	LOW	LOW	LOW	LOW	Study was terminated early due to meeting pre-defined criteria for efficacy.	LOW
Ehrmann – Canada	No	LOW	LOW	LOW	LOW	LOW	Study was terminated early due to meeting pre-defined criteria for efficacy.	LOW
Fralick (2022)	No	LOW	LOW	LOW	LOW	LOW	Stopped early for futility	LOW
Rampon (2022)	No	LOW	LOW	LOW	LOW	LOW	Unclear whether stopped early for futility or poor protocol adherence	LOW
Harris (2022)	No	SOME CONCERNS	SOME CONCERNS	LOW	LOW	LOW	Unclear whether stopped early or reported results at interim analysis	SOME CONCERNS
Hashemian (2021)	No	LOW	SOME CONCERNS	LOW	LOW	HIGH		HIGH

Jayakumar (2021)	No	LOW	SOME CONCERNS	LOW	LOW	LOW		SOME CONCERNS
Johnson (2021)	No	SOME CONCERNS	LOW	LOW	LOW	LOW	Stopped early for poor protocol adherence	SOME CONCERNS
Qian (2022)	No	HIGH	LOW	LOW	LOW	LOW		HIGH
Rosén (2021)	No	LOW	LOW	LOW	LOW	LOW	Stopped early for futility	LOW

eTable 5 – Secondary Outcome – Changes in oxygenation and respiratory rate with awake prone positioning

Study Author	Number	Oxygenation measure	Baseline Proning Group	Baseline Usual Care Group	Post-Prone positioning	Post Usual Care	Interval Assessed
Alhazzani 2022	400<br>(205 PRONING, 195 UC)	SpO2:FiO2<br><br>FiO2	132 (103-74)<br><br>70 (55 to 90)	136 (110-181)<br><br>70 (52-90)	Day 1: 136 (104-179)<br>Day 4: 184 (123-238)<br>Day 1: 70 (52-90)<br>Day 4: 50 (40-70)	Day 1: 134 (107-171)<br>Day 4: 171 (123-238)<br>Day 1: 70 (56-80)<br>Day 4: 60 (40-80)	24 h<br>4 days
Ehrmann 2021[a]	1121<br>(564 PRONING, 557 UC)	SpO2:FiO2<br>ROX Index<br>RR	145 ± 4<br>6.3 ± 0.2<br>24.6 ± 0.5	NR	167 ± 5<br>7.9 ± 0.2<br>22.9 ± 0.5	NR	30-60 min
Fralick 2022	248<br>(126 PRONING, 122 UC)	SpO2:FiO2	303 (261-336)	305 (267-339)	336 (216-438)[b]	336 (232-443)[b]	72 h
Hashemian 2021	75<br>(45 PRONING+NIV, 30 NIV)	PaO2/FiO2	Mild 233.1 ± 15.7<br>Moderate 138.4 ± 18.5<br>Severe 76.9 ± 13	Mild 213.4 ± 14.9<br>Moderate 150.7 ± 17.7<br>Severe 79.6 ± 13.3	Mild 261.4 ± 18.0<br>Moderate 174.1 ± 18.8<br>Severe 132.2 ± 19.9	Mild 247.3 ± 18.7<br>Moderate 178.5 ± 17.4<br>Severe 92.0 ± 17.4	24 h
Jayakumar 2021	60<br>(30 PRONING, 30 UC)	PaO2:FiO2	201.4 ± 118.8	185.6 ± 126.1	198.5 ± 87.6	171.7 ± 100.6	2 hours
Johnson 2021	30<br>(15 PRONING, 15 UC)	Change in PaO2:FiO2	NR	NR	-80.2 (-138.8 to -21.4)<br>-70.5 (-116.4 to -24.6)	-18.2 (-63.0 to 26.5)<br>-15.0 (-45.0 to 15)	72 hours<br>48 hours
Kharat 2021	27<br>(10 PRONING, 17 UC)	SpO2:FiO2<br><br>RR	318 (284-341)<br><br>22 (20-25.8)	336 (303-388)<br><br>20 (16-26)	390 (303-432)<br><br>20 (17.3-22.8)	336 (294-422)<br><br>20 (18-24)	24 hours
Qian 2022	501<br>(258 PRONING, 243 UC)	Maximum FiO2	NR	NR	Day 1: 45.32 ± 29.08)<br>Day 5: 40.59 ± 31.98	Day 1 40.36 ± 27.10<br>Day 5 37.10 ± 30.97	5 days
Taylor 2021	40<br>(27 PRONING, 13 UC)	Median nadir SpO2:FiO2	NR	NR	253 (192-269)	216 (147-294)	48 h

[a]Values extracted from figure using online software at https://pronings.automeris.io/wpd/

[b]Adjusted value for baseline SpO2:FiO2, age, sex

Abbreviations: FiO2, fraction of inspired oxygen; NIV, non-invasive ventilation; NR, not reported; PaO2/FiO2, arterial partial pressure of oxygen/fraction of inspired oxygen; ROX, respiratory rate-oxygenation; RR, respiratory rate; SpO2:FiO2, oxygen saturation to fraction of inspired oxygen ratio; UC, usual care

eTable 6 – Reported Adverse Events in Studies Examining Awake Prone Positioning in Non-intubated Patients with Hypoxemic Respiratory Failure due to COVID-19.

Study	Reported Adverse Events	Awake proning Group no. (%)	Control Group no. (%)
**Alhazzani** 2022	Pain or discomfort	16 (8)	0 (0)
	Accidental removal of vascular access	1 (1)	0 (0)
	Desaturation	4 (2)	0 (0)
	Hypotension	1 (1)	0 (0)
	Nausea	1 (1)	0 (0)
	Other	3 (2)	0 (0)
**Ehrmann** 2021[a]	Skin breakdown	8 (1)	10 (2)
	Vomiting	15 (3)	18 (3)
	Central or arterial line dislodgement	26 (5)	17 (3)
	Cardiac arrest	3 (1)	1 (0)
**Fralick** 2021	Aspiration pneumonia	2 (2)	1 (1)
	Venous thromboembolism	3 (2)	2 (2)
**Rampon** 2022	Positioning related discomfort	14 (9)	28 (21)
	Accidental removal of vascular access	9 (6)	12 (9)
	Accidental removal of urinary catheter	1 (0.6)	0 (0)
**Harris** 2022	Positioning related discomfort	0 (0)	1 (3)
**Hashemian** 2021	None reported	-	-
**Jayakumar** 2021	None reported	-	-
**Johnson** 2021	None reported	-	-
**Kharat** 2021	Positioning related discomfort	None reported	5 (50)
**Qian** 2022	None reported	-	-
**Rosén** 2021	Pressure sores	2 (6)	9 (23)
	Vomiting	1 (3)	0 (0)
	Central or arterial line dislodgement	0 (0)	0 (0)
	Cardiac arrest	2 (6)	1 (3)
**Taylor** 2021	Pressure sore	0 (0)	0 (0)
	Accidental removal of vascular access	1 (4)	0 (0)
	Emergent intubation outside of the ICU	0 (0)	0 (0)

a – Ehrmann et al prospective meta-analysis only reported pooled adverse events from all six included studies.

Abbreviations: ICU, intensive care unit

eTable 7 –Sensitivity analysis: Comparison of relative risk for primary outcome of intubation and secondary outcome of mortality using random effects with the DerSimonian and Laird approach and random effects with restricted maximum likelihood with Hartung-Knapp-Sidik-Jonkman correction.

**Outcome**	**Random Effects Model**<br>DerSimonian and Laird	**Random Effects Model**<br>Restricted maximum likelihood with Hartung-Knapp-Sidik-Jonkman correction
**Intubation**<br>Relative Risk (95% confidence interval)	0.83 (0.73 to 0.94)	0.83 (0.74 to 0.93)
**Mortality**<br>(Relative Risk, 95% confidence interval)	0.90 (0.76 to 1.07)	0.90 (0.79 to 1.03)

eFigure 1 – Funnel plot and Egger's test for the primary outcome of intubation

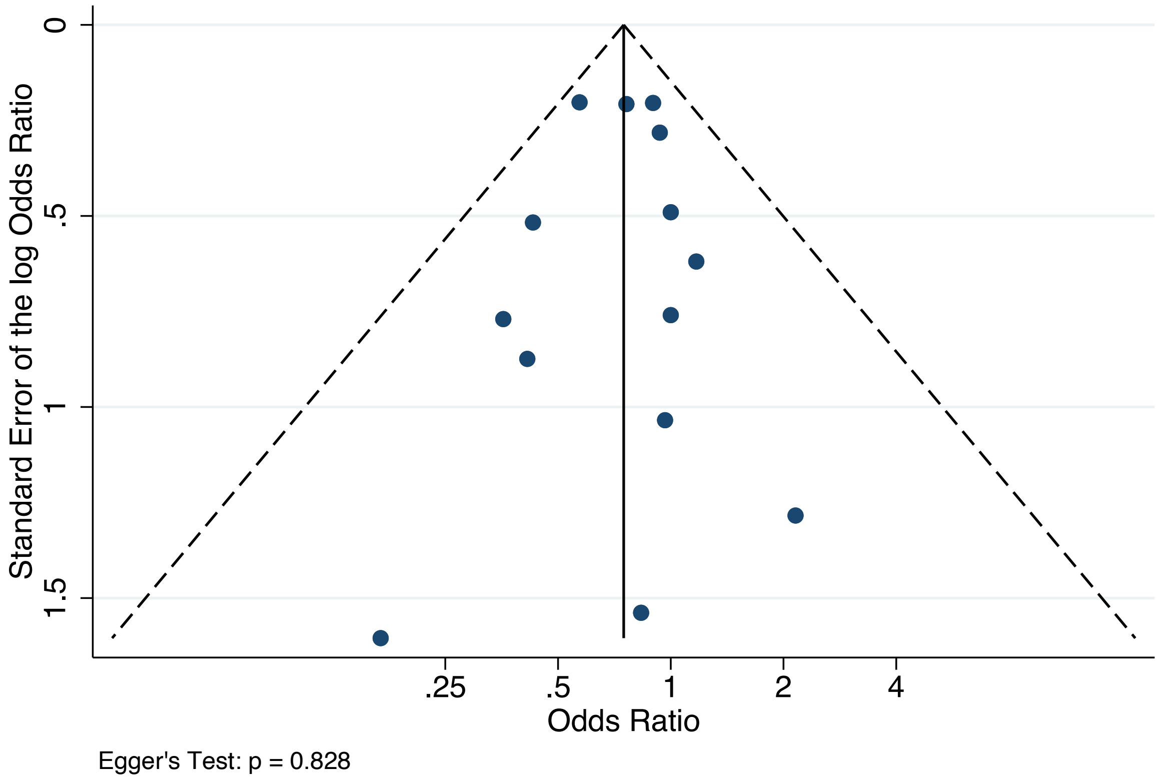

eFigure 2 – Funnel plot and Egger's test for secondary outcome of mortality

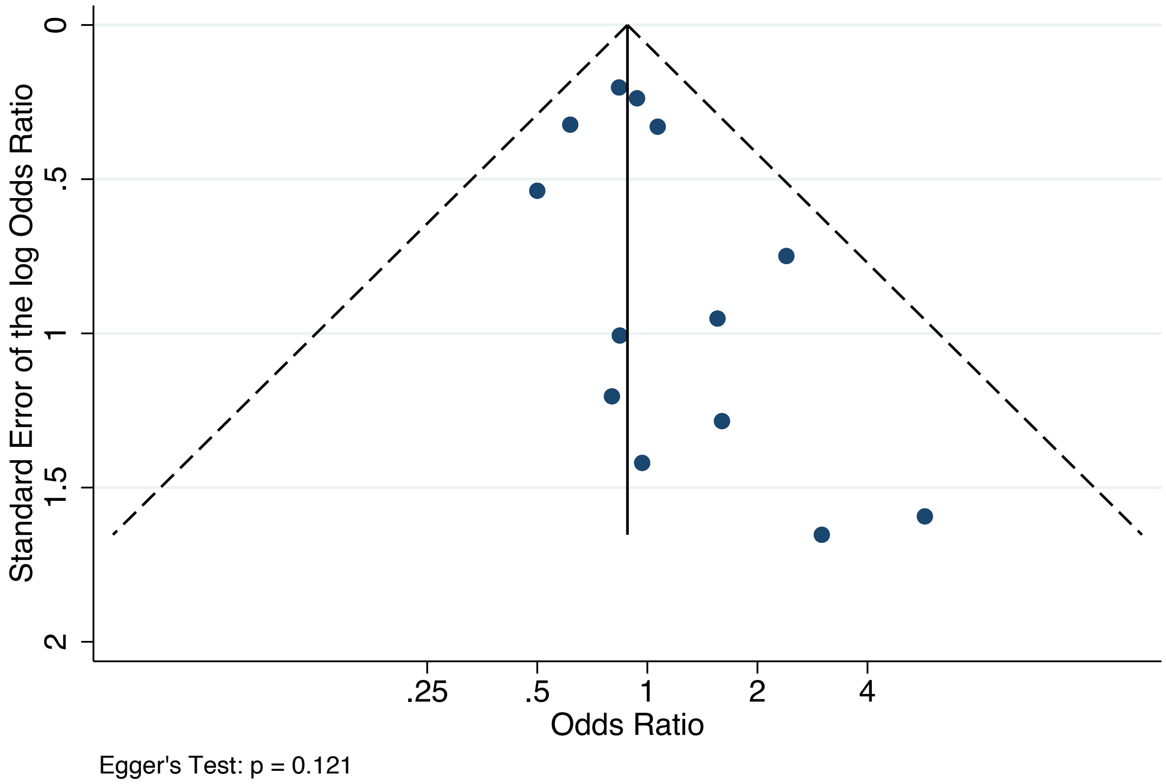

eFigure 3 – Secondary outcome: ventilator free days

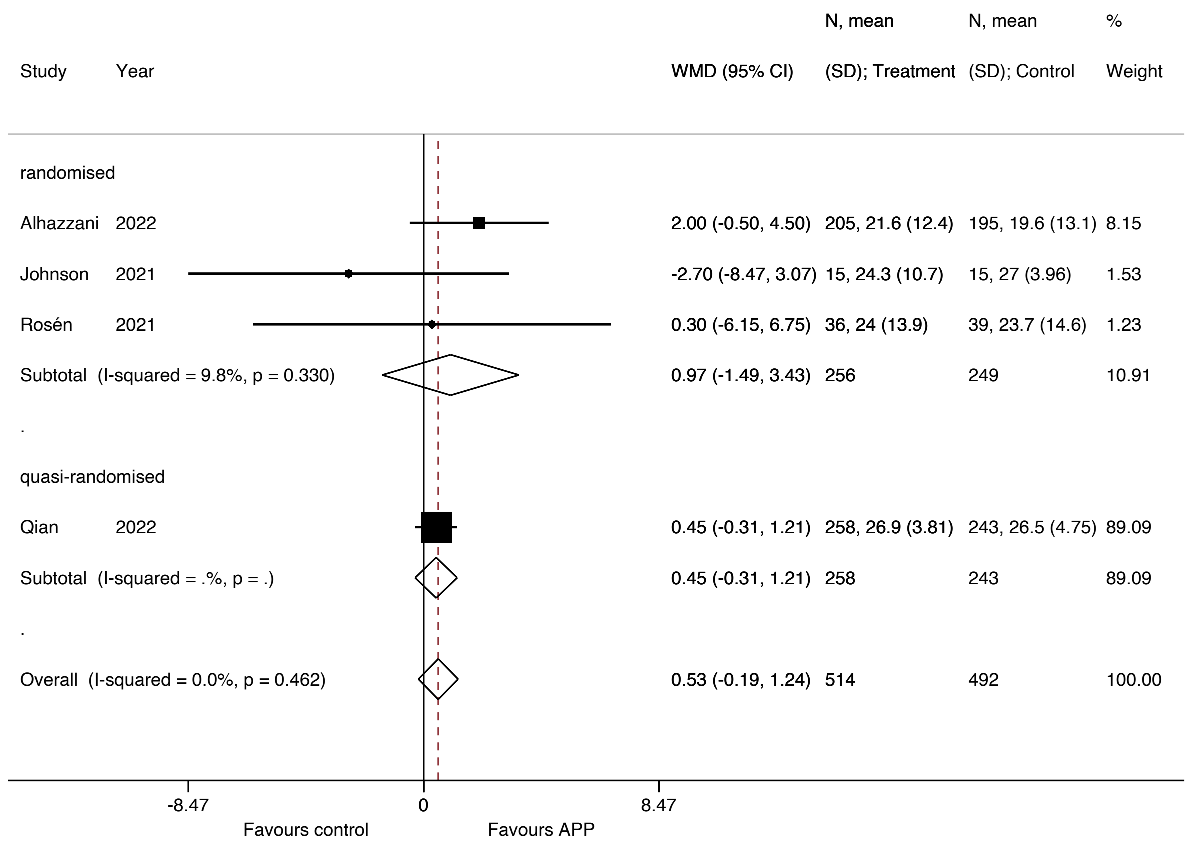

Three randomized trials and one quasi-randomized trial reported ventilator free days. All trials reported ventilator-free days at 30 days with the exception of one study (Qian) that reported ventilator free days at 28 days. The quasi-randomised trial (Qian) was stratified separately. Means and standard deviations were calculated from median and interquartile ranges as necessary according to Wan et al. (PMID 25524443). SD – standard deviation; WMD– weighted mean difference

eFigure 4 – Secondary outcome: intensive care unit length of stay

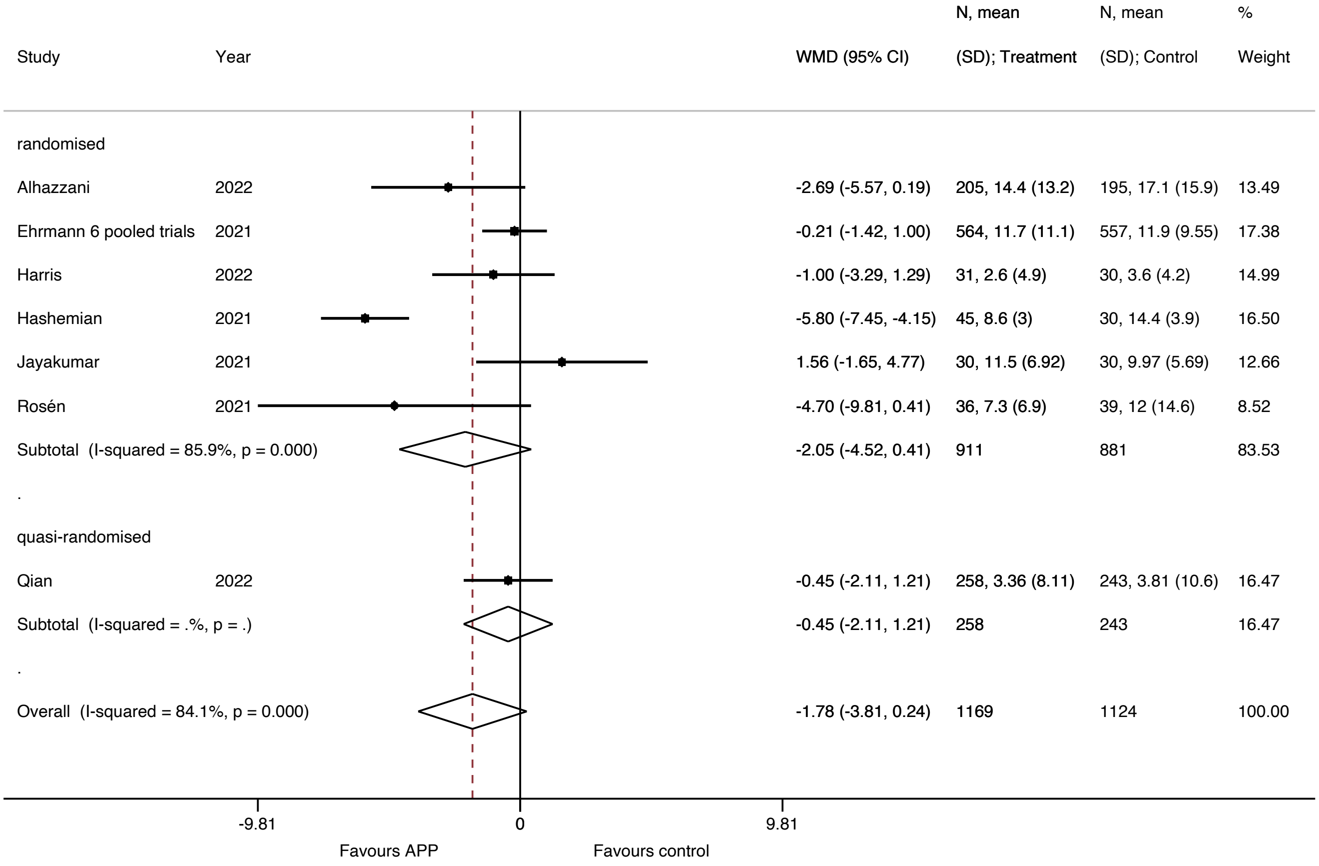

Intensive care unit length of stay (days) was reported in five individual trials (Alhazzani/Harris/Hashemian/Jayakumar/Rosén) and the Ehrmann et al. prospective meta-analysis, which reported pooled data from six individual trials. The quasi-randomised trial (Qian) also reported intensive care unit length of stay and was stratified separately. Individual trials in the Ehrmann et al. study could not be analyzed separately as the intensive care unit length of stay was not reported for each trial. Means and standard deviations were calculated from median and interquartile ranges as necessary according to Wan et al. (PMID 25524443). APP – awake prone positioning; SD – standard deviation; WMD – weighted mean difference.

eFigure 5 - Secondary outcome: hospital length of stay

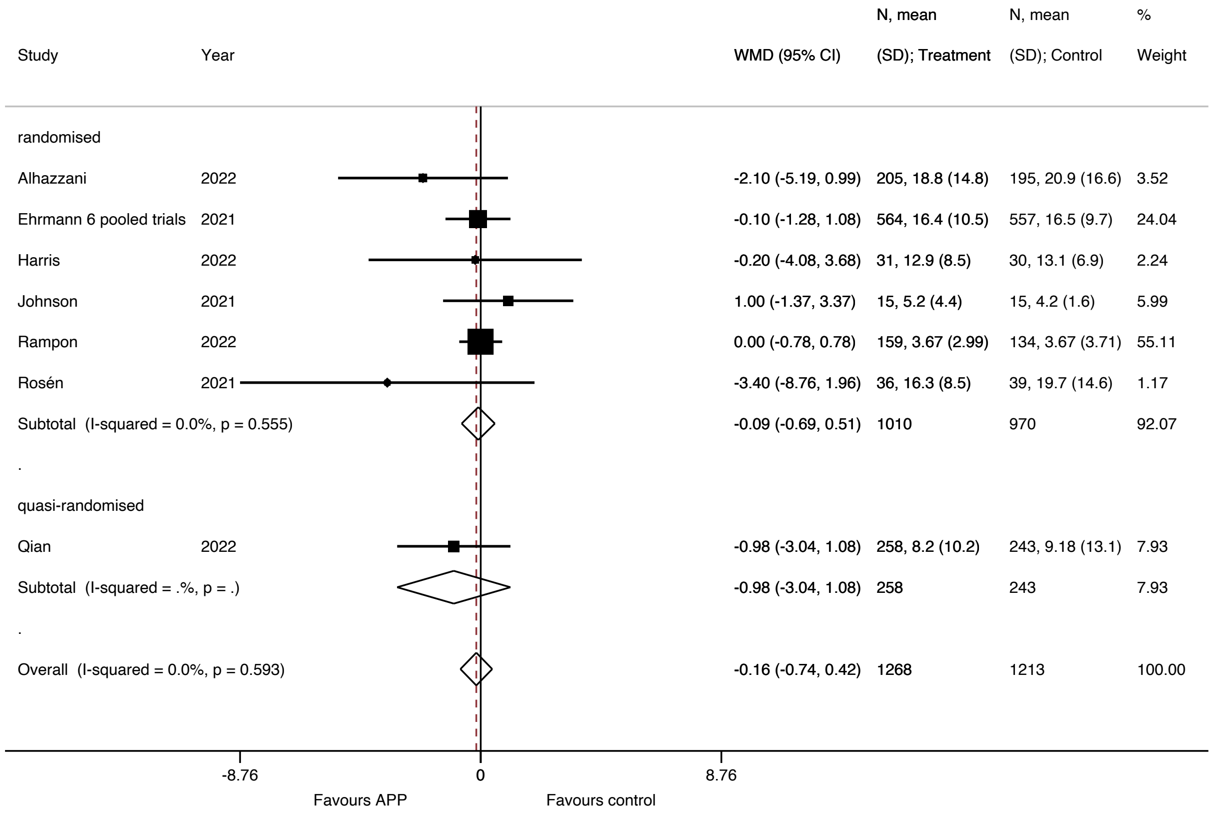

Hospital length of stay (days) was reported in five individual trials (Alhazzani/Rampon/Harris/Johnson//Rosén) and for the overall Ehrmann et al. prospective meta-analysis, which reported pooled data from six individual trials. Individual trials in the Ehrmann et al. study could not be analyzed separately as the hospital length of stay was not reported for each trial. The quasi-randomised trial (Qian) also reported hospital length of stay and was stratified separately. Two studies that were cluster randomized controlled trials (Taylor/Kharat) did not report the intra-cluster correlation coefficients and were excluded from this analysis. Means and standard deviations were calculated from median and interquartile ranges as necessary according to Wan et al. (PMID 25524443). APP – awake prone positioning; SD – standard deviation; WMD – weighted mean difference.

eFigure 6 - Secondary outcome: escalation of Oxygen Modality

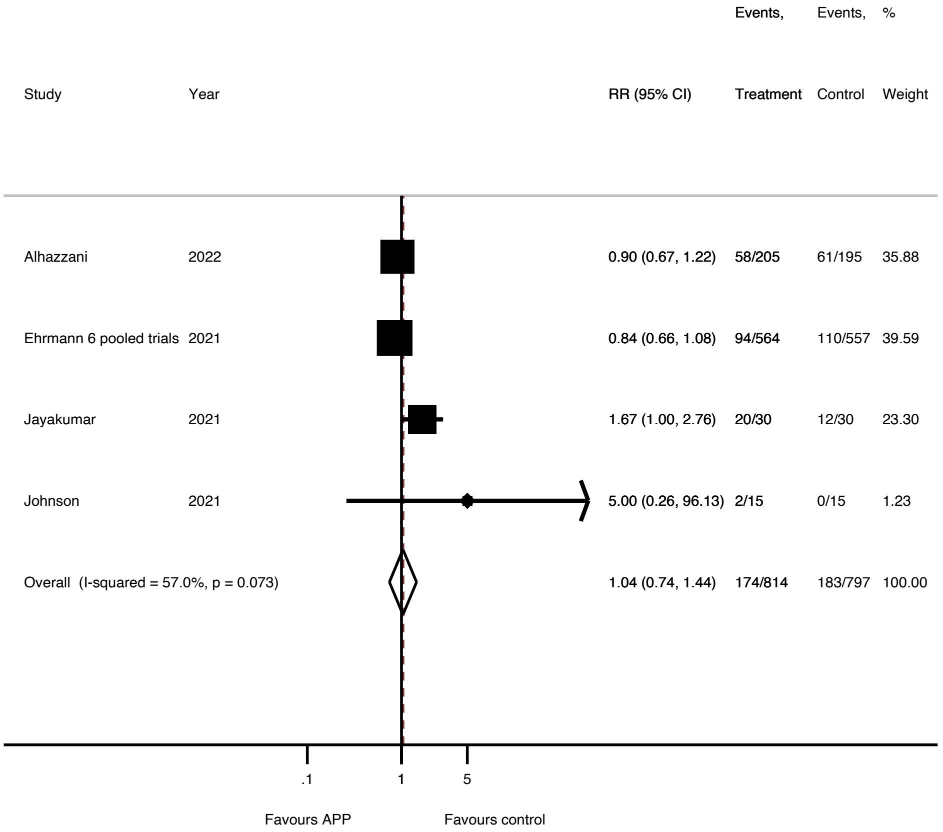

Three individual trials (Alhazzani/Jayakumar/Johnson) reported the proportion of patients who required escalation of oxygen modality (e.g. from low-flow to high-flow or non-invasive ventilation, or from high-flow to non-invasive ventilation) and for the overall Ehrmann et al. prospective meta-analysis, which reported pooled data from six individual trials. Individual trials in the Ehrmann et al. prospective meta-analysis could not be analyzed separately as the outcome was not reported for each trial individually. Abbreviations: RR = relative risk.

eFigure 7 – Bayesian trace, autocorrelation, histogram, and density plots for mean effect (theta) and between-study heterogeneity (tau2)

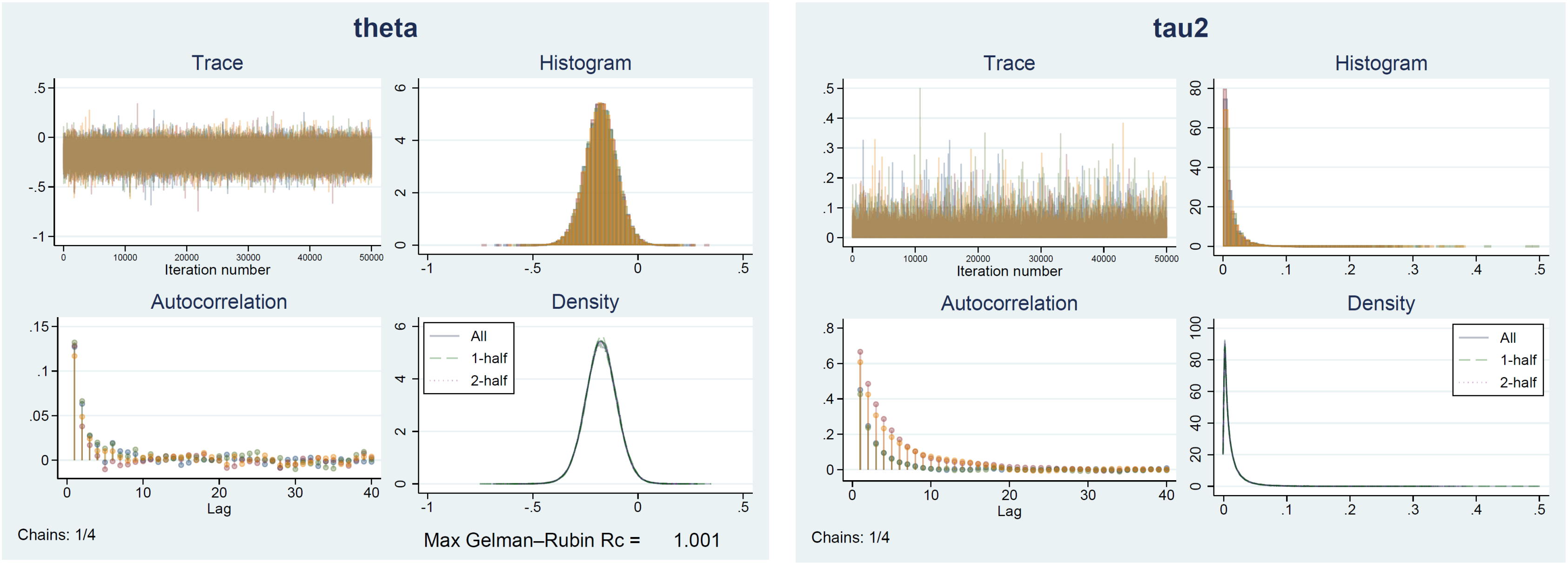

Efficiency was typically 20% and acceptance 45%. Gelman-Rubin statistics were all less than 1.1 (typically around 1.002) and consistent with good mixing seen in visual inspection of trace, autocorrelation, histogram and density plots.

eFigure 8 - Trial sequential analysis for the primary outcome of endotracheal intubation

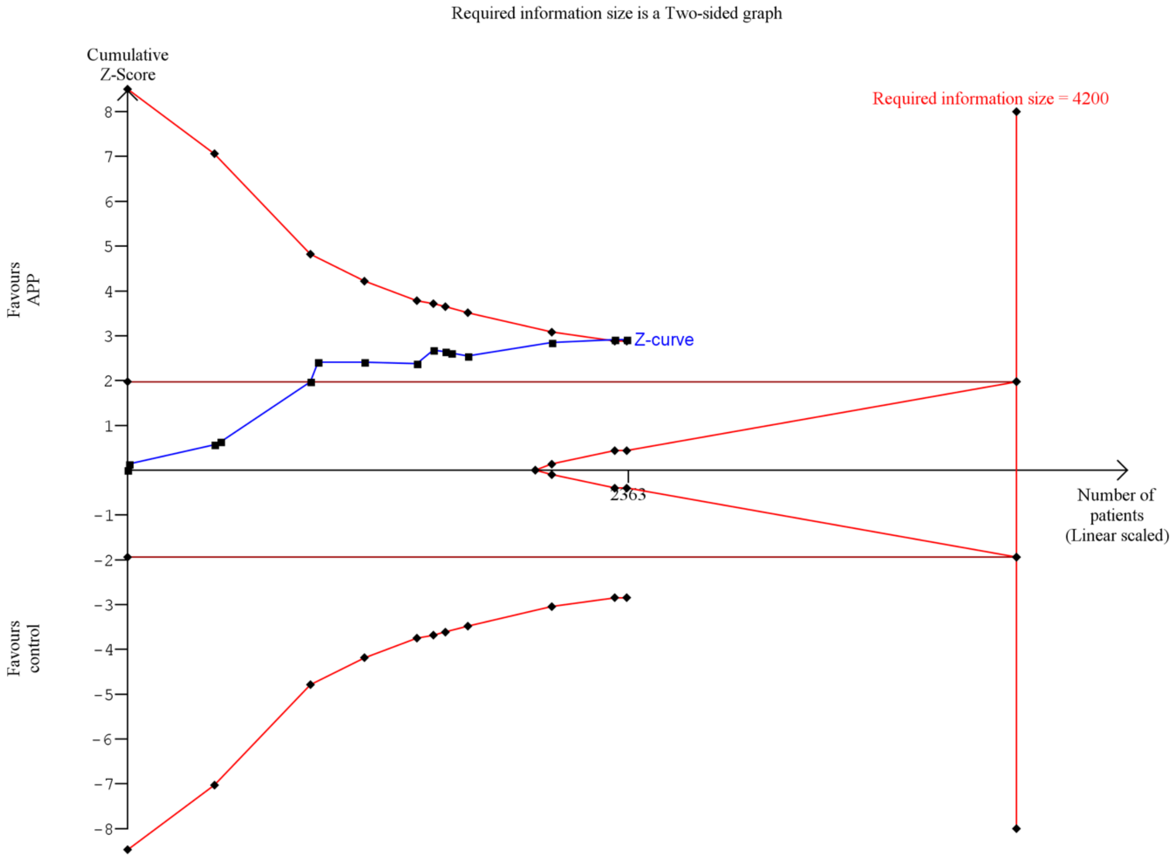

Endotracheal intubation trial sequential analysis (TSA). The TSA includes only randomised controlled trials and excluded the quasi-randomized trial by Qian et al. Settings: alpha 5%, power 90%, Relative risk reduction (RRR) 15%, control event proportion (CEP) 29.8% (343/1152), model variance-based heterogeneity correction, relative risk (RR), random effects model (DerSimonian & Laird). Accrued information size (AIS) / required information size (RIS): 2363 / 4200 (56.3%). The TSA-adjusted confidence interval (CI): RR 0.83 (0.70 to 0.99).

eFigure 9 – Trial sequential analysis for the secondary outcomes

A. Mortality

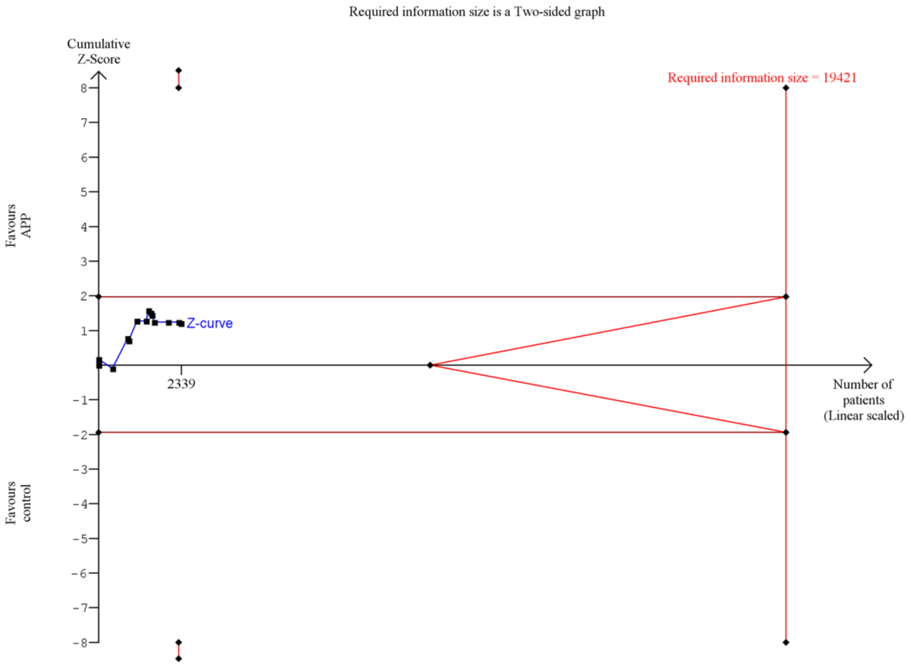

B. Ventilator-free days

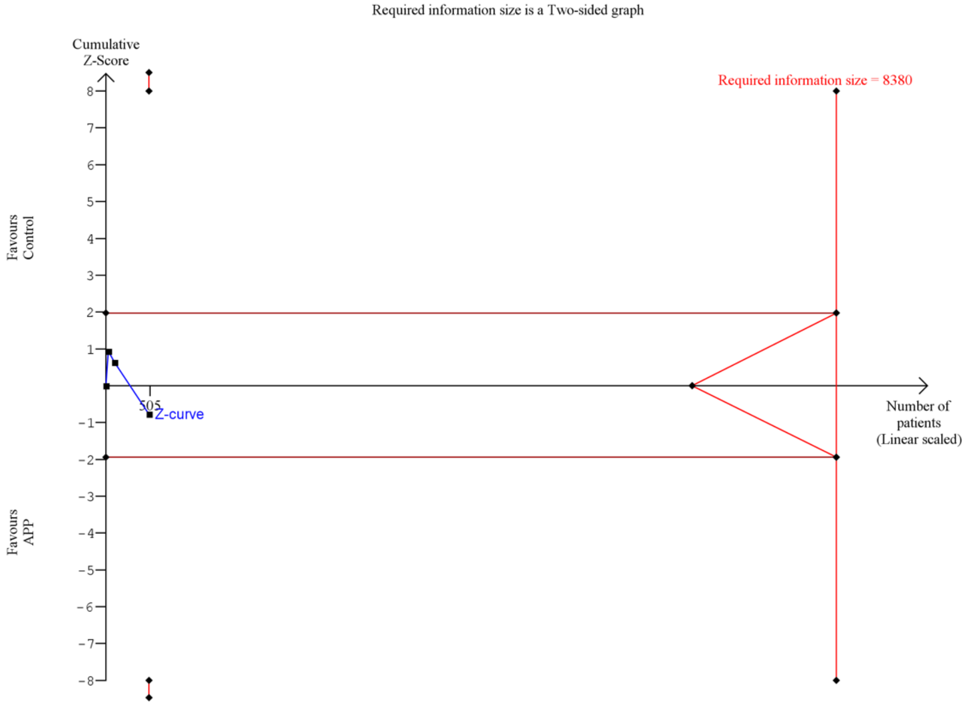

C. Intensive care unit length of stay

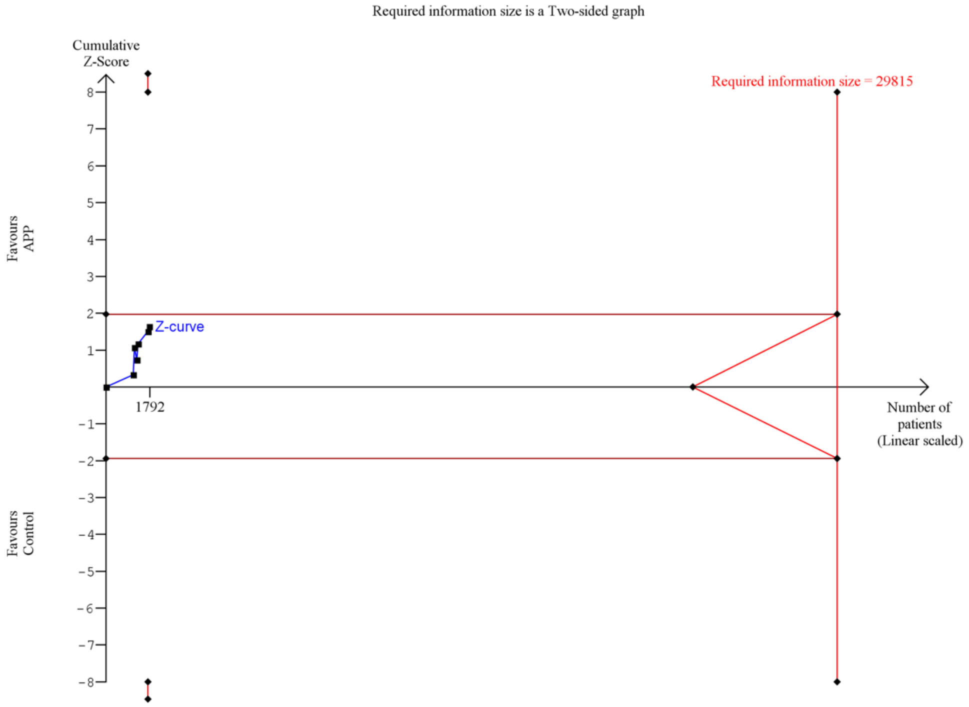

D. Hospital length of stay

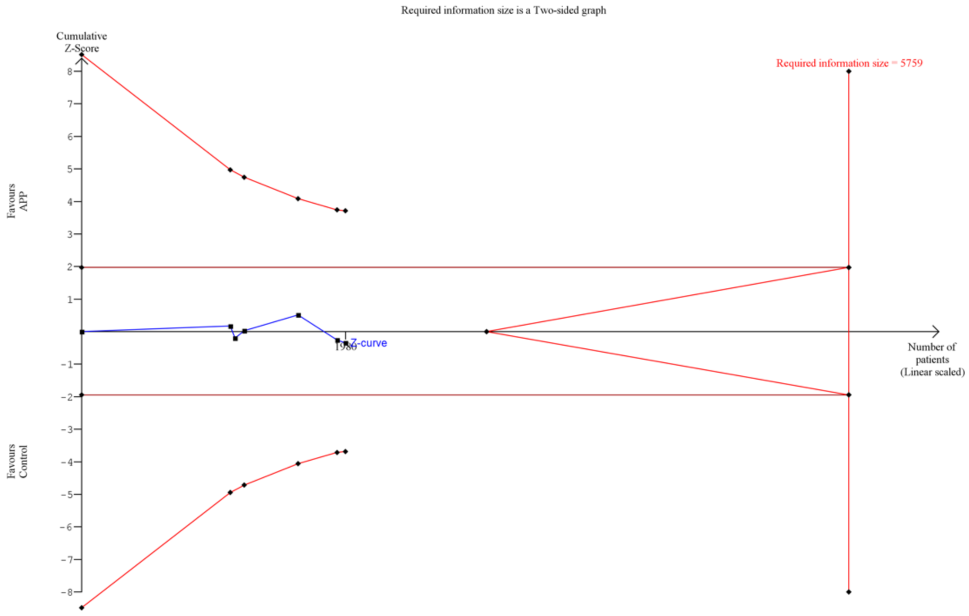

The trial sequential analyses for secondary outcomes include only randomised controlled trials and excluded the quasi-randomized trial by Qian et al.

A. Mortality trial sequential analysis (TSA). Settings: alpha 5%, power 90%, relative risk reduction (RRR) 10%, control event proportion (CEP) 17.2% (196/1140), model variance-based heterogeneity correction, relative risk (RR), random effects model (DerSimonian & Laird). Accrued information size (AIS) / Required information size (RIS): 2339 / 19421 (12.0%). TSA-adjusted confidence interval (CI): RR 0.90 (0.45 to 1.82).
B. Ventilator-free days TSA. Settings: alpha 5%, power 90%, effect size of interest mean difference (MD) 1 day increase, variance based on the included trials, model variance-based heterogeneity correction, MD, random effects model (DerSimonian & Laird). AIS / RIS: 505 / 8381 (6.0%). TSA-adjusted CI: MD 0.97 (-9.08 to 11.02).
C. Intensive care unit length of stay TSA. Settings: alpha 5%, power 90%, effect size of interest MD 1 day decrease, variance based on the included trials, model variance-based heterogeneity correction, MD, random effects model (DerSimoninan & Laird). AIS / RIS: 1792 / 29815 (6.0%). TSA-adjusted CI: MD -2.06 (-12.12 to 8.01).
D. Hospital length of stay TSA. Settings: alpha 5%, power 90%, effect size of interest MD 1 day decrease, variance based on the included trials, model variance-based heterogeneity correction, MD, random effects model (DerSimoninan & Laird). AIS / RIS: 1980 / 5759 (34.4%). TSA-adjusted CI: MD 0.19 (-1.76 to 2.15).

eFigure 10 – Sensitivity analysis for intubation excluding unpublished studies

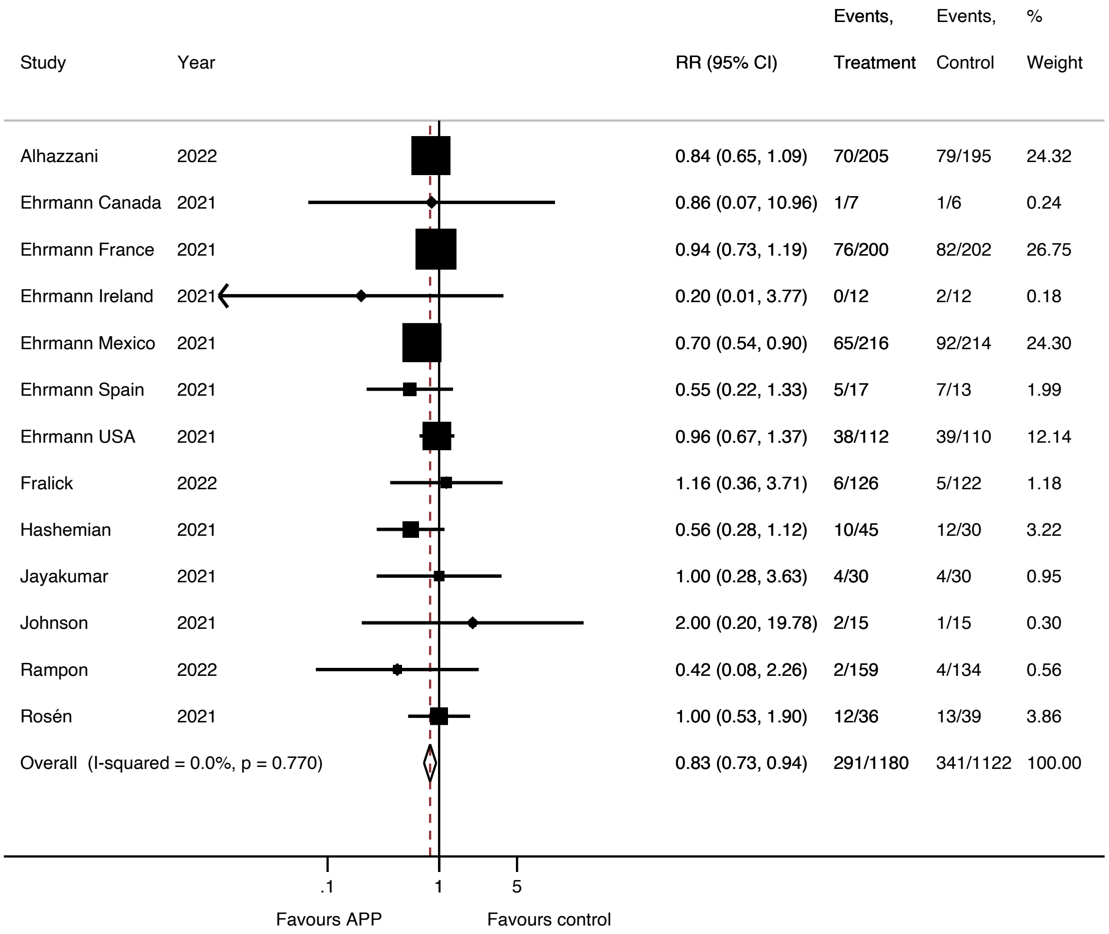

Thirteen randomised trials were pooled. One unpublished trial that was reported in a previous meta-analysis was excluded in this analysis (Harris) because the individual trial results were not published in a peer-reviewed journal. Two trials were published but had no intubation events in both arms (Taylor/Kharat) and were excluded from this analysis. The quasi-randomised trial (Qian) was also excluded. Abbreviations: RR = relative risk

eFigure 11 – Sensitivity analysis for intubation excluding studies at risk of bias

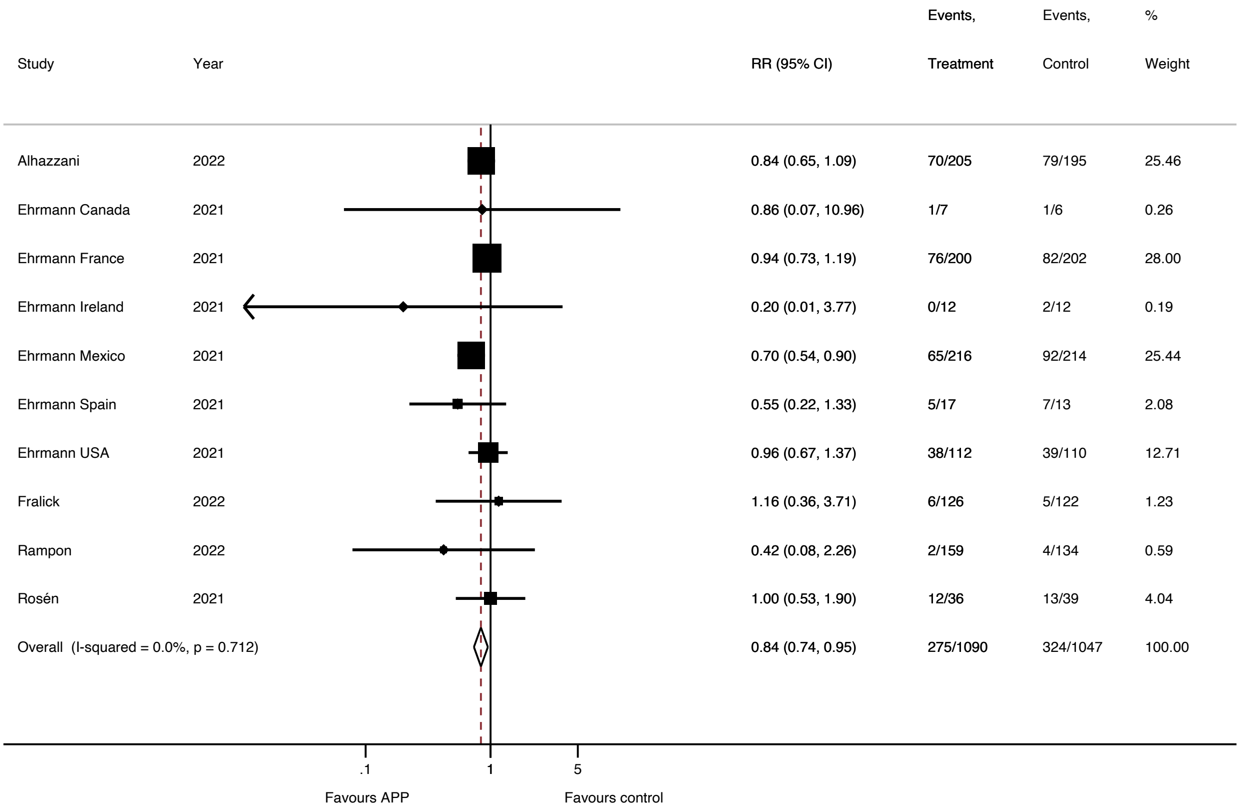

Ten trials were pooled. Two trials were excluded from this analysis due to high risk of bias (Qian, Hashemian) and three were excluded due to some risk of bias (Harris, Jayakumar, Johnson). Two trials that had no intubation events in both arms (Taylor/Kharat) were also excluded from this analysis.

Abbreviations: RR = relative risk

eFigure 12 – Sensitivity analysis for intubation excluding trials that were stopped early

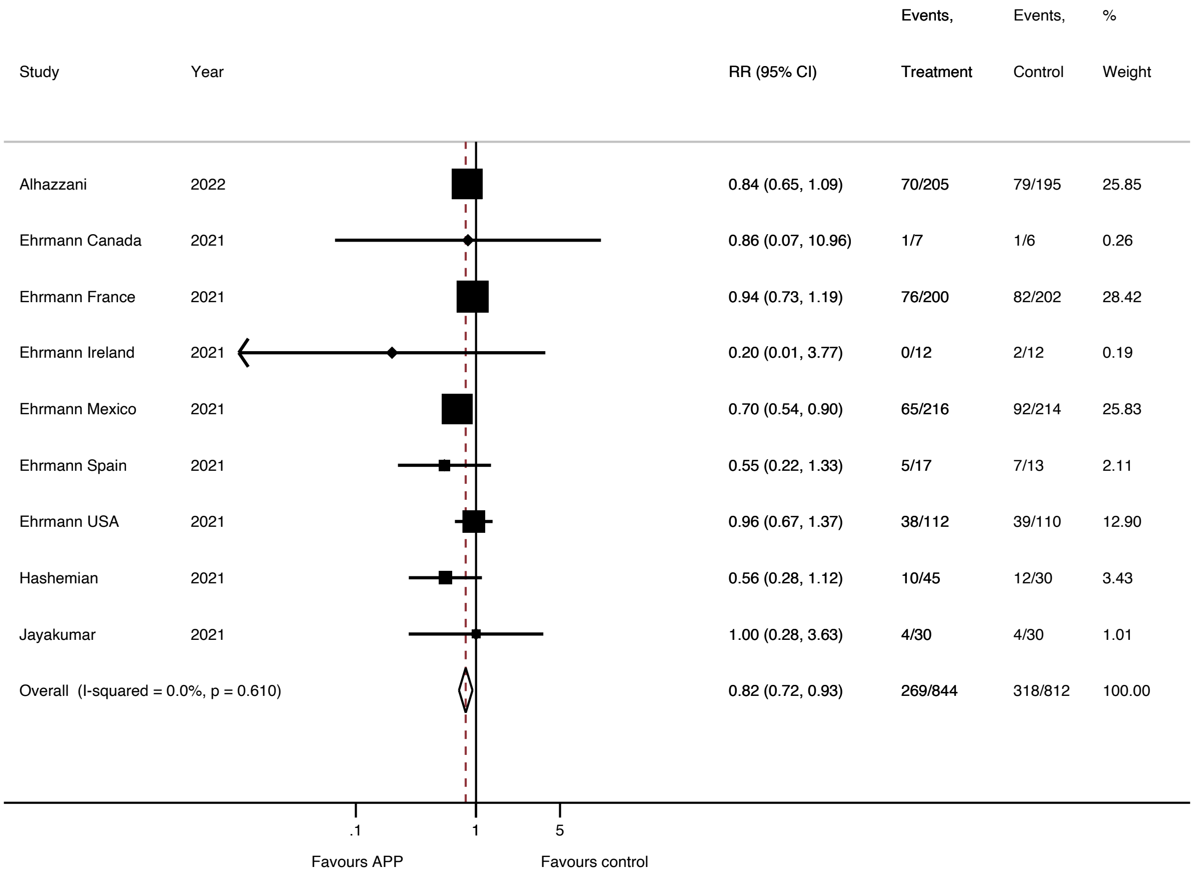

Nine trials were pooled. Two trials were excluded due to stopping early for futility (Fralick/Rosen), one trial due to slow enrollment (Rampon), and one trial was excluded due to stopping early for poor adherence to the protocol (Johnson). The Ehrmann et al. prospective meta-analysis of six trials was stopped after an interim analysis demonstrated efficacy. At the time the interim analysis was complete, the prospective meta-analysis had reached its target sample size and was included in this analysis. One trial was excluded as it was an unpublished study reported identified in a meta-analysis (Harris) with fewer patients than the registered sample size (NCT04853979). Two trials that had no intubation events in both arms (Taylor/Kharat) were also excluded from this analysis. The quasi-randomised trial (Qian) was also excluded. Abbreviations: RR = relative risk

eFigure 13 – Sensitivity analysis for endotracheal intubation outcome using pooled data from the Ehrmann et al. prospective meta-analysis

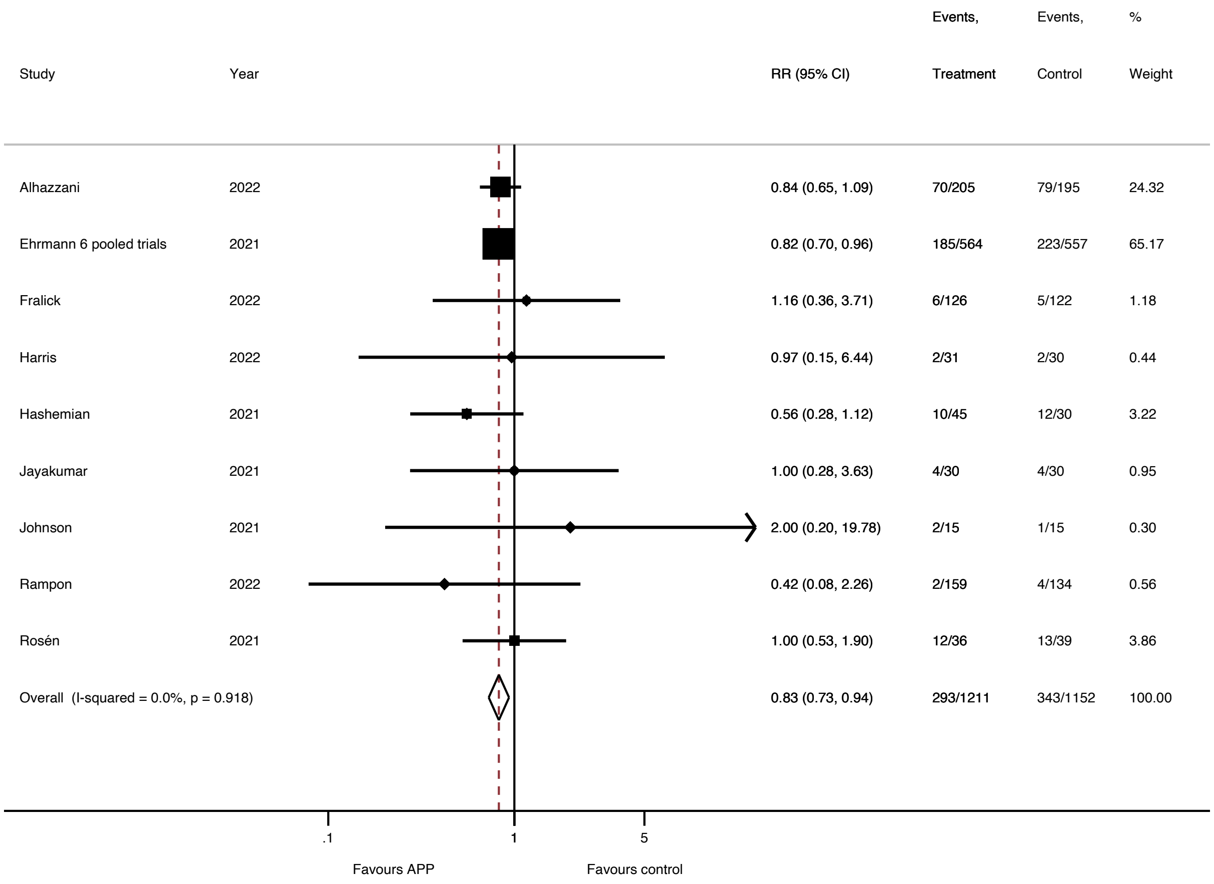

Nine trials were pooled. The pooled data from the prospective meta-analysis of 6 trials were considered as a single study, as reported by Ehrmann et al. Two trials that had no intubation events in both arms (Taylor/Kharat) were excluded from this analysis. The quasi-randomised trial (Qian) was also excluded. Abbreviations: RR = relative risk

eFigure 14 – Sensitivity analysis for intubation excluding trials with no events in either arm

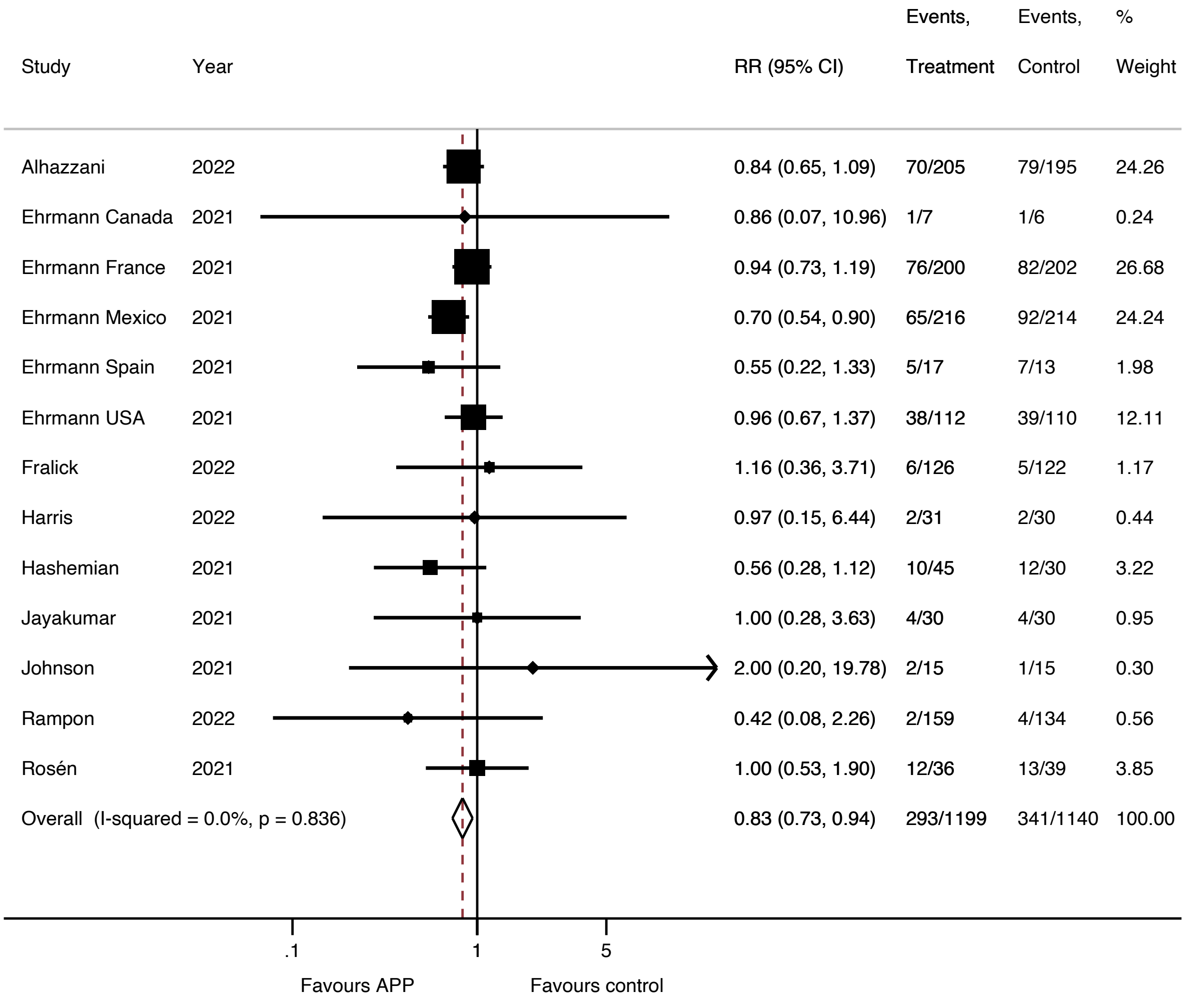

Thirteen trials were pooled. Two trials that had no intubation events in both arms (Taylor/Kharat) were excluded from this analysis and one trial with no intubation events in the proning arm was also excluded (Ehrmann – Ireland). The quasi-randomised trial (Qian) was also excluded. Abbreviations: RR = relative risk

eFigure 15 –Sensitivity analyses for intubation and mortality outcomes including a trial with quasi-randomized allocation

A) Endotracheal Intubation Outcome

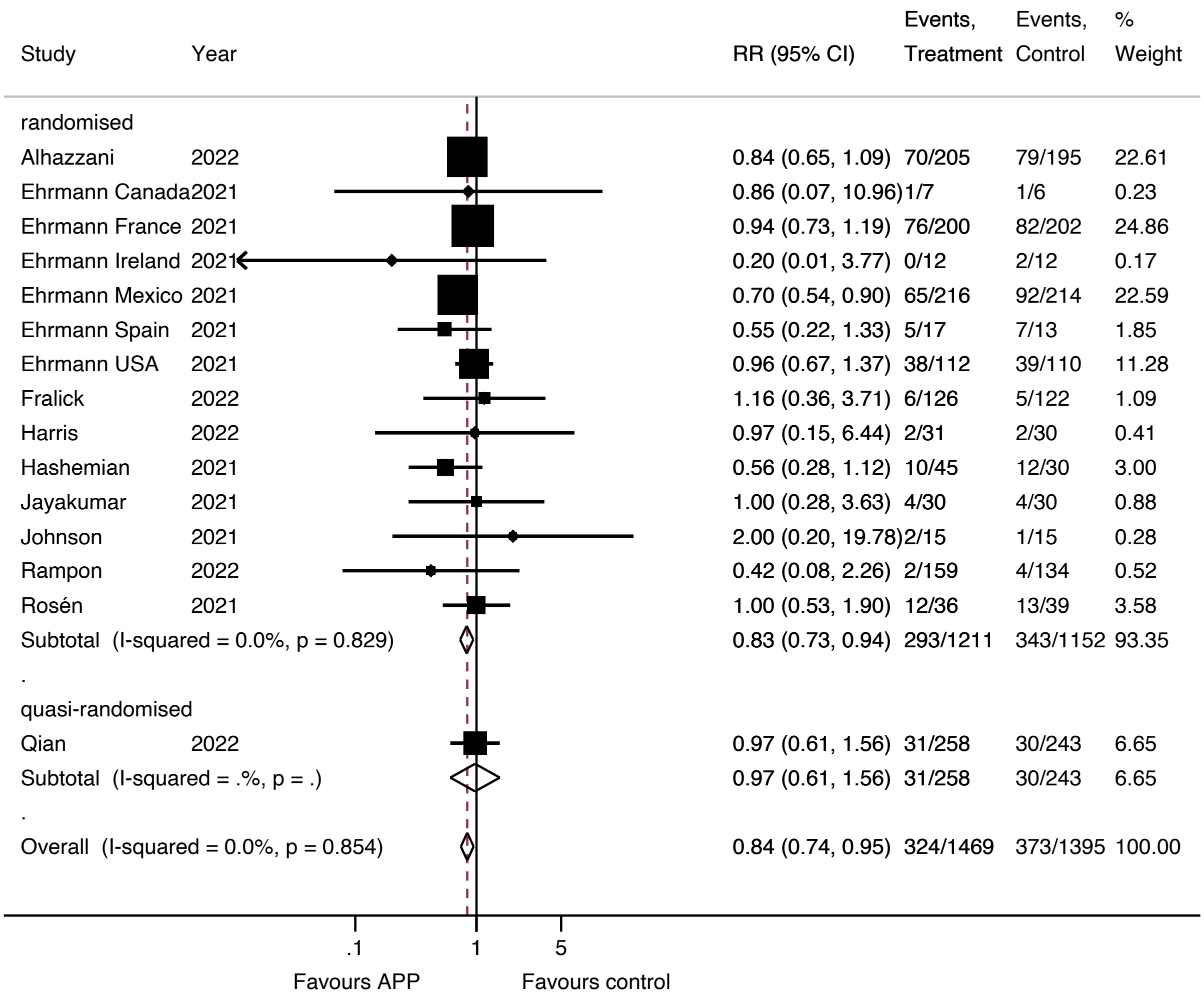

B) Mortality Outcome

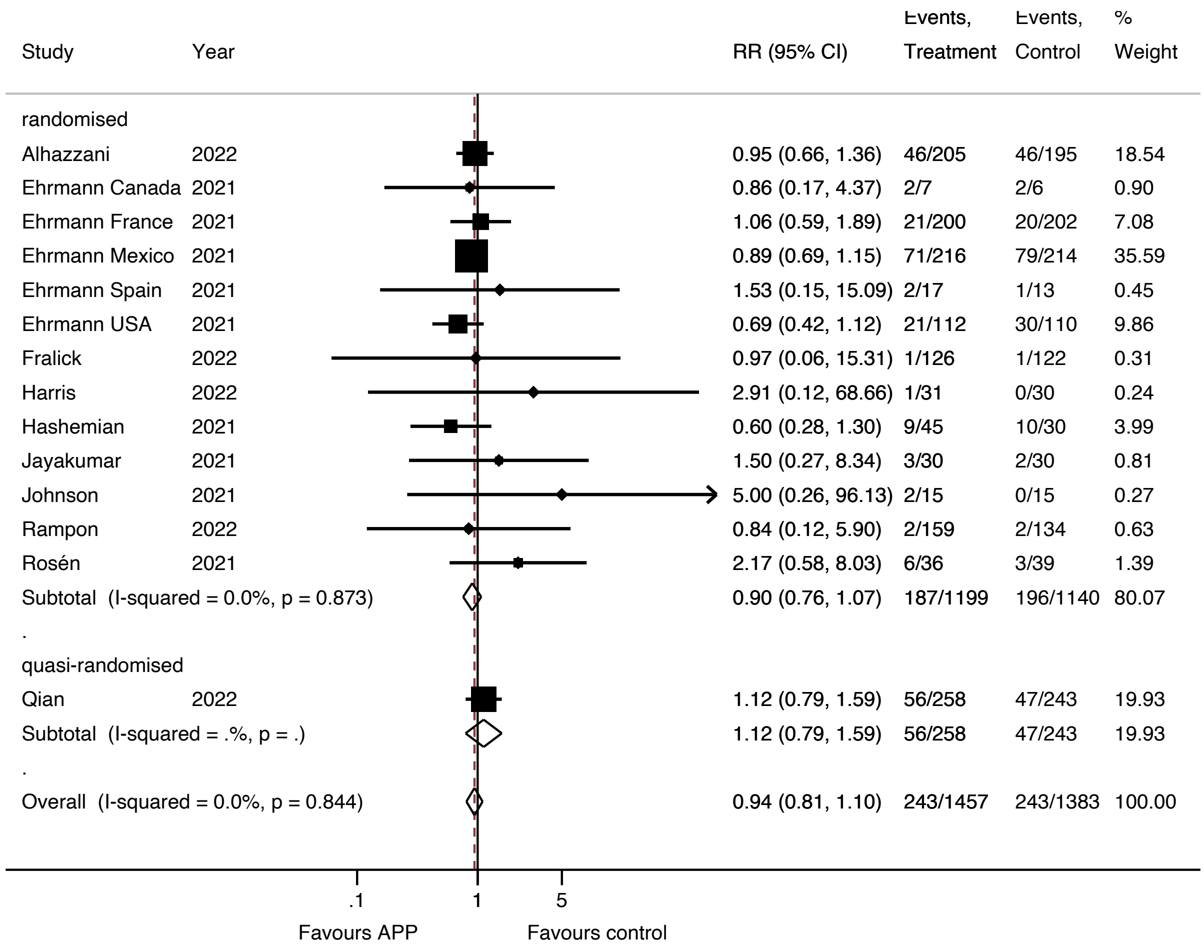

A. Fifteen trials were pooled. The trial with quasi-randomized allocation was included (Qian). Two trials that had no intubation events in both arms (Taylor/Kharat) were also excluded from this analysis. Qian et al reported ever intubated during the hospital stay.

B. Fourteen trials were pooled. The trial with quasi-randomized allocation was included (Qian). Three trials that had no mortality events in both arms (Taylor/Kharat/Ehrmann - Ireland) were also excluded from this analysis. Qian et al reported mortality at 28-day hospital mortality.

Abbreviations: RR = relative risk

eFigure 16 – Meta-regression of median duration of prone positioning in the intervention group and intubation

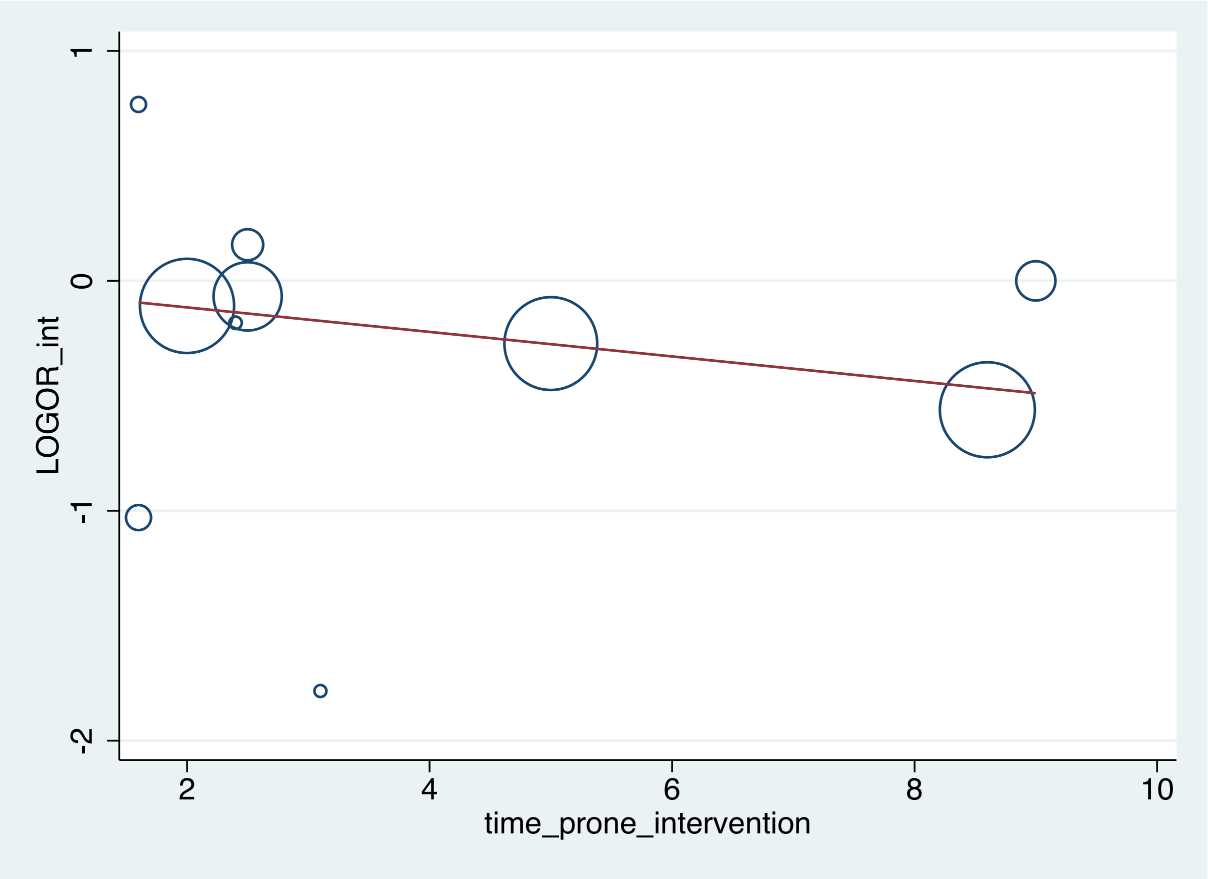

Meta-regression of the association between the log odds ratio (dependent variable) and the median duration of prone positioning (independent variable) in the intervention group of each trial. Ten trials were included in this analysis. Two trials that had no intubation events in both arms (Taylor/Kharat) and were excluded from this analysis. Four trials did not report median or mean duration of prone positioning and were excluded from this analysis (Jayakumar, Harris, Rampon, Hashemian). The quasi-randomised trial (Qian) was also excluded. Abbreviation: LOGOR _INT = log odds ratio of intubation; time_prone_intervention = median duratiof of proning in each trial.
